# A phase II, multicenter, single-arm study of pemigatinib in patients with metastatic or unresectable colorectal cancer harboring FGFR alterations

**DOI:** 10.1093/oncolo/oyaf069

**Published:** 2025-06-14

**Authors:** Margaret C Wheless, Tyler J Zemla, Joleen M Hubbard, John H Strickler, Olumide B Gbolahan, Luke Wilson, Blake Waechter, Fang-Shu Ou, Andrew B Nixon, Tanios S Bekaii-Saab, Kristen K Ciombor

**Affiliations:** Department of Internal Medicine, Division of Hematology/Oncology, Vanderbilt University Medical Center, Nashville, TN, United States; Department of Quantitative Health Sciences, Mayo Clinic, Rochester, MN, United States; Allina Health Cancer Institute, Rochester, MN, United States; Department of Medicine, Division of Medical Oncology, Duke University Medical Center Durham, NC, United States; Department of Hematology and Medical Oncology, Emory University School of Medicine, Atlanta, GA, United States; Department of Quantitative Health Sciences, Mayo Clinic, Rochester, MN, United States; Department of Quantitative Health Sciences, Mayo Clinic, Rochester, MN, United States; Department of Quantitative Health Sciences, Mayo Clinic, Rochester, MN, United States; Department of Medicine, Division of Medical Oncology, Duke University Medical Center Durham, NC, United States; Division of Medical Oncology and Hematology, Mayo Clinic, Scottsdale, AZ, United States; Department of Internal Medicine, Division of Hematology/Oncology, Vanderbilt University Medical Center, Nashville, TN, United States

**Keywords:** FGF, FGFR, colorectal cancer, pemigatinib

## Abstract

**Background:**

FGFR alterations are known to be driver alterations in several tumor types. We aimed to assess the efficacy of pemigatinib, an oral FGFR1-3 inhibitor, in patients with metastatic or unresectable colorectal cancer whose tumors harbored FGF/FGFR alterations.

**Patients and Methods:**

The ACCRU-GI-1701 is a single-arm phase II trial which enrolled patients with previously treated FGF/FGFR-altered metastatic colorectal cancer to receive oral pemigatinib daily in 21-day cycles. The primary endpoint is objective response. Secondary endpoints include clinical benefit, progression-free survival, overall survival, quality of life, and adverse events (AEs). This trial was registered with ClinicalTrials.gov (NCT04096417).

**Results:**

Of the 14 patients included in the interim analysis, the objective response rate as well as clinical benefit rate were 0%. Given these results, the trial closed to enrollment after stage one due to futility. A total of 42.9% of patients had at least one grade 3 or higher AE, the most common being anemia and fatigue.

**Conclusion:**

Pemigatinib monotherapy did not lead to objective responses in patients with chemorefractory metastatic colorectal cancer harboring FGF/FGFR alterations, although it was overall relatively well tolerated with no new safety signals. Notably, 93% (*n* = 13) of patients had only FGF/FGFR mutations and amplifications; one patient had an FGFR3-WHSC1 fusion at a low cfDNA percentage (0.02%).

**Implications for Practice**

Pemigatinib monotherapy did not lead to objective responses in patients with chemorefractory metastatic colorectal cancer (mCRC) harboring FGF/FGFR alterations. Pemigatinib’s efficacy in targeting FGFR fusions in mCRC patients remains unknown because only one patient with an FGFR fusion with low percentage of cell-free DNA (cfDNA) in the blood was included in this study. Further characterization of FGF/FGFR alterations in mCRC is needed, including evaluating effects of downstream RAS/RAF mutations, VEGF inhibition, and treatment history in patients harboring FGF/FGFR alterations.

## Introduction

Colorectal cancer remains the third most common cause of cancer-related mortality in males and the fourth most common cause of cancer-related mortality in females in the United States.^1^ Though mortality has decreased by 2% since 2012 due to advances in treatment, colorectal cancer-related mortality remains a worldwide issue with a lifetime risk estimated at 5%.^2,3^ Unfortunately, up to 25% of patients present with advanced disease and 25%-50% of patients who present with localized disease will ultimately develop metastasis. The 5-year survival rate for metastatic colorectal cancer is estimated at just 12.5%, prompting the need for innovative treatment advances for these patients.^4^ For patients with disease refractory to standard chemotherapy, overall survival (OS) continues to decrease with subsequent lines of treatment.^5-7^ With the advent of tumor genomic sequencing, discovery of oncogenic driver mutations and the development of targeted agents has become an attractive therapeutic option.

Pemigatinib, an oral tyrosine kinase inhibitor, blocks fibroblast growth factor receptor 1, 2, and 3 (FGFR1-3) from binding its ligand FGF to prevent downstream signaling.^8^ FGFR has been implicated in several cellular proliferation and angiogenic pathways activating MAPK, PI3K, JAK/STAT, PLC signaling cascades, promoting epithelial to mesenchymal transformation, and fostering an immunosuppressive tumor microenvironment.^9-11^ Several mechanisms of FGF dysregulation have been recognized including amplifications, activating mutations, and fusions/translocations. Amplification results in increased FGFR expression causing ligand-independent FGFR activation, while fusion proteins result in dimerized (activated) receptors lacking negative feedback mechanisms, and activating mutations culminate in constitutively active FGFR.^8,12^

Dysregulation of the FGF pathway has been implicated as a driver alteration in several tumor types including cholangiocarcinoma, melanoma, breast, urothelial, gastric, prostate, and lung cancer,^10,12-14^ and FGFR inhibition has been successful in vitro and in vivo to inhibit cellular proliferation.^14-21^*FGFR* mutations have been reported in 5% of colon cancer, amplifications in 2%, and fusions in 0.7%, and are associated with a worse prognosis.^8,22^*FGFR2* amplification found in NCI-H716 colorectal cells derived from ascites of patients with poorly differentiated colon adenocarcinoma led to increased FGFR2 activation in vitro and in vivo which was required for downstream effector activity.^23^ It is postulated that FGF activation serves as an escape mechanism after treatment with either vascular endothelial growth factor receptor (VEGFR) inhibitors or epidermal growth factor receptor (EGFR) inhibitors to support invasion and angiogenesis.^24-26^ Increased FGF2 expression was found in 43 patients with colon cancer after treatment with bevacizumab, a VEGF inhibitor,^6,12^ and represents a treatment opportunity for genomically selected patients.

Pemigatinib has been approved for patients with FGFR fusion positive tumors, specifically in hematologic malignancies, cholangiocarcinoma, and other genomically selected advanced solid tumors.^27,28^ The phase I/II FIGHT-101 trial evaluated the recommended dose and efficacy of pemigatinib across multiple solid tumor types, including colon cancer. The efficacy of pemigatinib was most pronounced in patients with FGFR fusions or rearrangements and prompted the exploration of this targeted therapy in patients with solid tumors that harbor FGFR alterations.^28^ The ACCRU-GI-1701 phase II study aimed to evaluate the efficacy of pemigatinib in patients with chemorefractory metastatic or unresectable colorectal cancer harboring FGFR alterations.

## Methods

### Eligibility

Patients ≥ 18 years old with a histologically or cytologically confirmed diagnosis of metastatic or unresectable colorectal cancer harboring an activating FGF/FGFR1-3 alteration (gain of function mutation, translocation, or amplification) based on tumor tissue and/or blood testing were enrolled. Patients had progressed on or were intolerant to the following treatments, if eligible: fluoropyrimidine, oxaliplatin, irinotecan, pembrolizumab, nivolumab, an anti-VEGF monoclonal antibody, and an anti-EGFR monoclonal antibody. Other inclusion criteria included measurable disease per RECIST 1.1, ECOG performance status of 0-2, ANC ≥ 1500/mm^3^, platelets ≥ 100 000/mm^3^, hemoglobin ≥ 9 g/dL, total bilirubin ≤ 1.5× upper limit of normal (or ≤ 2.5× upper limit of normal if Gilbert's syndrome present), AST and ALT ≤ 2.5× the upper limit of normal (or ≤ 5× if the patient had liver metastases), normal serum phosphate, normal serum calcium, normal potassium levels, and serum creatinine ≤ 1.5× the upper limit of normal or a glomerular filtration rate of at least 30 mL/min. Exclusion criteria included known hypersensitivity to an FGFR inhibitor, retinal disorder, major surgery within 28 days of registration, brain or spinal metastases, significant gastrointestinal disorders that could affect absorption of pemigatinib, and use of potent CYP3A4 inhibitors or inducers. Full inclusion and exclusion criteria can be found in the Supplementary Protocol.

### Study design and intervention

ACCRU-GI-1701 is a single-arm, phase II study designed to assess the efficacy of pemigatinib in patients with chemorefractory metastatic colorectal cancer. After screening and enrollment, eligible patients were assigned to receive oral pemigatinib 13.5 mg daily for 21 days. If patients tolerated the first cycle without grade 2 or higher AEs or hyperphosphatemia >5.5 mg/dL, pemigatinib was increased to 18 mg daily for subsequent cycles.

### Monitoring and dose reductions

Response to therapy was evaluated every 9 weeks with computed tomography or magnetic resonance imaging using RECIST 1.1 guidelines. Laboratory monitoring with blood samples was drawn at baseline, on cycle 1 days 1, 8, and 15, and then with each cycle from cycle 2 onward. Eye examinations were required on screening and on the last day of treatment, and as clinically indicated. The pemigatinib dose could be modified for any reported toxicities to 13.5 mg daily (if escalated to 18 mg) or further decreased to 9 mg daily (see Supplementary Protocol for full dosing modification guidelines). Hyperphosphatemia, a known on-target effect, was treated initially with a low phosphate diet. If phosphate levels were between >7 mg/dL and ≤ 10 mg/dL, phosphate-binding therapy was initiated for 2 weeks and if no improvement was seen in phosphate levels, pemigatinib could be held for up to 2 weeks. If phosphate levels were >10 mg/dL, labs were monitored bi-weekly and pemigatinib could be held and resumed at a reduced dose when phosphate levels were <7 mg/dL. Adverse events (AEs) were assessed using CTCAE v5.0 grading at each visit.

### Endpoints

The primary endpoint was objective response rate, which is defined as a complete or partial response per RECIST 1.1, without a confirmatory scan. Secondary endpoints include clinical benefit, progression-free survival (PFS), OS, quality of life (QOL), and AEs. Clinical benefit is defined as achieving an objective response or having sustained stable disease for at least 36 weeks. Progression-free survival is defined as the time from registration to disease progression (per RECIST 1.1) or death, whichever occurs first. Patients without an event are censored at their last disease evaluation. Overall survival is defined as the time from registration to death due to any cause. Patients are censored at the time they were last known to be alive. Quality of life outcomes are measured using the linear analogue self-assessment (LASA) overall QOL score.

### Study design

Patients who were eligible, consented, received any protocol treatment, and without any major treatment violations were evaluable for the primary endpoint. A Simon two-stage minimax design was used. The largest success proportion where the proposed treatment regimen would be considered ineffective in this population was 5%, and the smallest success proportion that would warrant subsequent studies with the proposed regimen in this patient population was 20%. At the first stage, if at least one success was observed in the first 12 evaluable patients, the study would continue to stage 2. If no responses were observed, the treatment would be deemed ineffective in this patient population and the study would be terminated. If the study proceeded to stage 2, an additional 9 evaluable patients would be enrolled. If 3 or more successes were observed in the first 21 evaluable patients, we would recommend further testing of this regimen in subsequent studies in this population. Given our null success proportion is 0.05 and our alternative success proportion is 0.2, assuming a type 1 error rate of 0.1, a sample size of 21 evaluable patients provides 82% exact power to detect a true ORR of 20% or greater. Since there were no objective responses in the first 12 evaluable patients, the study was terminated for futility after stage one. This trial was registered with ClinicalTrials.gov (NCT04096417).

### Statistical analysis

Patient characteristics were reported descriptively using mean, median, and range for continuous variables as well as count with percentage for categorical variables. Objective response rate, which is defined as the percentage of patients, among evaluable patients, who experienced an objective response, was calculated along with a 95% binomial CI. Clinical benefit rate (CBR) was calculated as the percentage of evaluable patients who experienced clinical benefit along with a 95% CI. For both PFS and OS, medians along with the corresponding 95% CI were calculated using the Kaplan-Meier Method. For QOL analysis, the mean and standard deviation of absolute change from baseline to the best on-treatment score was calculated among patients who have both baseline score and at least one on-treatment score. The best on-treatment score was defined as the largest QOL score (scale of 1-10) reported, while the patient was on treatment. Frequencies and percentages of all AEs reported on the trial were summarized. The Grade 3+ and Grade 4 rates were reported for all patients.

## Results

### Baseline characteristics

A total of 14 patients with metastatic colorectal cancer were enrolled between September 15, 2020 and January 18, 2022 and treated with pemigatinib. The median age was 61 years (range, 36-87), 10 (71.4%) of the patients were male, and the most common race was White (13, or 92.9%). All patients had an ECOG performance status of 0 (*n* = 7) or 1 (*n* = 7) (Table 1). Nine patients (64.3%) had left-sided tumors, while two (14.3%) had right-sided/transverse colon primaries. Four patients had been on regorafenib previously, 3 on trifludirine/tipiracil (TAS-102), and 1 patient had been on both agents. All patients had alteration(s) in FGF/FGFR1-3 based on either tissue and/or blood-based testing done at a Clinical Laboratory Improvement Amendments-certified laboratory (Table 2). No patients were excluded for primary endpoint analysis based on ineligibility or major violation after registration. Blood-based FGFR1 amplification was the most common alteration (*n* = 8/9, 88.9%) followed by tissue-based FGFR1 alteration (*n* = 3/13, 23.1%) and tissue-based FGFR2 mutation (*n* = 2/13, 15.4%) (Table 2). Blood-based FGFR3-WHSC1 fusion was detected in one patient with a cfDNA percentage of 0.02%. Eleven (78.6%) patients were KRAS wildtype, 12 (85.7%) were NRAS wildtype, and 12 (85.7%) were BRAF wildtype by NGS from tissue or blood or both.

**Table 1.** Baseline demographics.

Characteristic	Total (*N* = 14)
Age: mean, median, (range) in years	58.9, 60.5, (36.0-87.0)
Race, *n* (%)	
White	13 (92.9%)
Not reported	1 (7.1%)
Sex, *n* (%)	
Female	4 (28.6%)
Male	10 (71.4%)
Ethnicity, *n* (%)	
Not Hispanic or Latino	12 (85.7%)
Not reported	2 (14.3%)
ECOG Performance Status, *n* (%)	
0	7 (50.0%)
1	7 (50.0%)
Disease Characteristics, *n* (%)	
Metastatic	14 (100.0%)
Prior Treatment, *n* (%)	
Trifluridine/tipiracil (TAS-102)	3 (21.4%)
Regorafenib	4 (28.6%)
Both TAS-102 and regorafenib	1 (7.1%)
Neither TAS-102 no regorafenib	6 (42.9%)
Primary Tumor Site, *n* (%)	
Left	9 (64.3%)
Right/Transverse	2 (14.3%)
Other	1 (7.1%)
Multiple primaries	2 (14.3%)
KRAS Status, *n* (%)	
Mutation	2 (14.3%)
Wildtype	11 (78.6%)
Unknown	1 (7.1%)
NRAS Status, *n* (%)	12 (85.7%)
Wildtype	2 (14.3%)
Unknown	
BRAF Status, *n* (%)	
Mutation	1 (7.1%)
Wildtype	12 (85.7%)
Unknown	1 (7.1%)
Discrete Number of Metastatic Sites, *n* (%)	
1	4 (28.6%)
2+	10 (71.4%)

**Table 2.** FGF/FGFR alteration type.

Alteration type	Total (*N* = 14)
Tissue-based FGFR1, *n* (%)	
Amplification	1 (7.7%)
Mutation	2 (15.4%)
No alteration	10 (76.9%)
Missing	1
Tissue-Based FGFR2, *n* (%)	
Mutation	2 (15.4%)
No alteration	11 (84.6%)
Missing	1
Tissue-Based FGFR3, *n* (%)	
Amplification	1 (7.7%)
Mutation	1 (7.7%)
No alteration	11 (84.6%)
Missing	1
Tissue-Based FGFR Other, *n* (%)	
Amplification	1 (7.7%)
Mutation	1 (7.7%)
No alteration	11 (84.6%)
Missing	1
Tissue-Based FGF Gene Alteration, *n* (%)	
Yes	3 (23.1%)
No	10 (76.9%)
Missing	1
Blood-Based FGFR1, *n* (%)	
Amplification	8 (88.9%)
No alteration	1 (11.1%)
Missing	5
Blood-Based FGFR2, *n* (%)	
Mutation	1 (11.1%)
No alteration	8 (88.9%)
Missing	5
Blood-Based FGFR3, *n* (%)	
Translocation/fusion	1 (11.1%)
No alteration	8 (88.9%)
Missing	5
Blood-Based FGFR Other, *n* (%)	
Mutation	1 (11.1%)
No alteration	8 (88.9%)
Missing	5
Blood-Based FGF Gene Alteration, *n* (%)	
No	9 (100.0%)
Missing	5

### Primary and secondary outcomes

**Figure 1.**
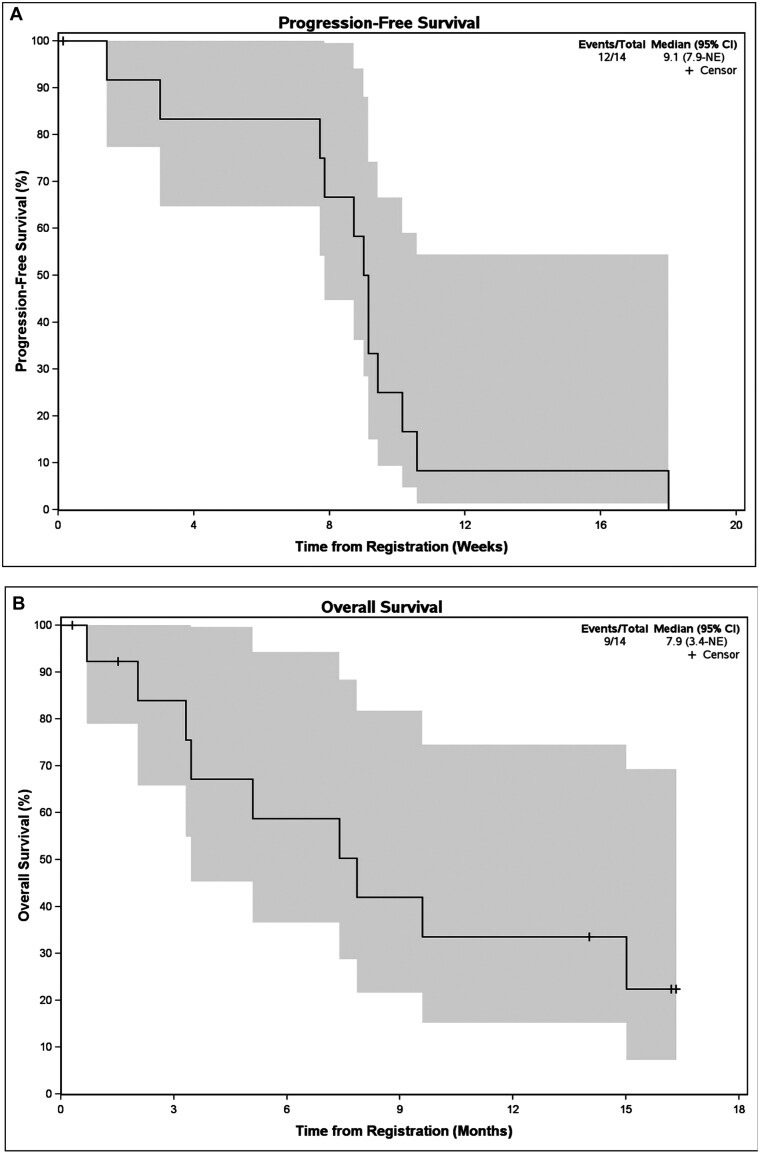
Progression-free survival (PFS) and overall survival (OS): (A) PFS demonstrating median PFS of 9.1 weeks (95% CI, 7. 9-NE) and (B) OS revealing median OS of 7.9 months (95% CI, 3.4-NE).

Of 14 patients who were evaluable for response, 9 patients had progressive disease as best response, 1 patient had stable disease, and 4 patients came off protocol treatment prior to their first re-staging scan yielding an ORR and CBR of 0% (95% CI, 0%-23.2%). Median PFS was 9.1 weeks (95% CI, 7.9-not evaluable, NE) and mOS was 7.9 months (95% CI, 3.4-NE), shown in Figure 1A and B, respectively. Patients received an average of 2.5 cycles (range: 1-6 cycles) of pemigatinib. Of the 14 patients, 2 were escalated to 18 mg daily dose for cycle 2. QOL measurements were evaluable in 11 patients indicating no statistically significant changes in QOL while on treatment, ie, mean change from baseline to best on-treatment LASA Overall QOL of –0.1 with an SD of 1.22.

### Adverse events

Of all evaluable patients, 6 had at least one grade 3 or higher AE and 2 had grade 4 or higher AEs. Neither of the 2 patients escalated to the higher pemigatinib dose had grade 3 or 4 AEs. The most common grade 1/2 AEs were hyperphosphatemia (*n* = 9), AST elevation (*n* = 8), alkaline phosphatase elevation (*n* = 8), anemia (*n* = 8), fatigue (*n* = 6), diarrhea (*n* = 5), ALT elevation (*n* = 5), and anorexia (*n* = 5). The most common grade 3 AEs included anemia (*n* = 2) and fatigue (*n* = 2). Other clinically relevant grade 3 AEs included hyperphosphatemia, alkaline phosphatase elevation, diarrhea, blood bilirubin increased, vomiting, gastrointestinal fistula, lung infection, lymphopenia, and pain (all *n* = 1). There was one grade 4 AE (hydrocephalus) and one grade 5 AE (second malignancy) which were at least possibly related to pemigatinib treatment. Selected AEs clinically relevant to pemigatinib administration are shown in Table 3.

**Table 3.** Selected adverse events.

Selected adverse events	Grade 1 (*N* (%))	Grade 2 (*N* (%))	Grade 3 (*N* (%))	Grade 4 (*N* (%))
Nausea	3 (21.4)	1 (7.1)	0	0
Oral Mucositis	1 (7.1)	2 (14.3)	0	0
Platelet Count Decreased	2 (14.3)	1 (7.1)	0	0
Vomiting	2 (14.3)	0	1 (7.1)	0
Anemia	6 (42.9)	2 (14.3)	2 (14.3)	0
Hyperphosphatemia	5 (35.7)	4 (28.6)	1 (7.1)	0
Fatigue	5 (35.7)	1 (7.1)	2 (14.3)	0
Alkaline phosphatase increased	7 (50.0)	1 (7.1)	1 (7.1)	0
Diarrhea	2 (14.3)	3 (21.4)	1 (7.1)	0
Anorexia	3 (21.4)	2 (14.3)	0	0
Blood bilirubin increased	3 (21.4)	1 (7.1)	1 (7.1)	0
Dehydration	0	2 (14.3)	0	0
Dysgeusia	0	1 (7.1)	0	0

One patient experienced a grade 4 event (hydrocephalus), and another patient experienced a grade 5 event (second malignancy) that were deemed unrelated to pemigatinib treatment.

## Discussion

Pemigatinib did not show clinical efficacy in this cohort of patients with chemorefractory, metastatic colorectal cancer with FGF/FGFR amplifications, fusions, and mutations. No patients achieved an objective response from treatment. Median PFS and OS were 9.1 weeks and 7.9 months, respectively. There were no new safety concerns associated with pemigatinib, and 6 patients (42.9%) had grade 3 or higher AEs, the most common being fatigue and anemia.

The majority of patients enrolled (93%, *n* = 13) had either FGF/FGFR mutations or amplifications and so it is unknown if pemigatinib would be efficacious in mCRC patients with FGF/FGFR fusions or translocations. Though one patient had an FGFR3-WHSC1 fusion, this was only detected in the blood at a low cfDNA percentage of 0.02%. TACC3 is the most common fusion partner for FGFR3, but several novel fusion partners have been recently identified, including WHSC1 (also known as NSD2 or MMSET).^29,30^ FGFR3 fusions are more commonly found in urothelial carcinoma, glioblastoma, and nonsmall cell lung cancer and have not been reported in CRC so its clinical significance remains unknown.^31^

The FIGHT-202 study led to the approval of pemigatinib in cholangiocarcinoma based on its efficacy in patients with FGFR2 fusions and rearrangements (*N* = 146; fusions/rearrangements, *n* = 107). The objective response in the FGFR2 fusion/rearrangement cohort was 35.5% including 50 patients (46.7%) with SD, 35 (32.7%) with PR, and 3 (2.8%) with PD. Of the 20 patients with FGF/FGFR alterations that were not fusions or rearrangements, the best response seen was SD in 8 patients (40%). The mOS of 21.1 months (95% CI, 14.8-NE) in the FGFR2 fusion/rearrangement group was significantly longer than the FGF/FGFR alteration group (mOS 6.7; 95% CI, 2.1-10.6).^32^ Similarly, the phase 2 FIGHT-203 trial evaluated the efficacy of pemigatinib in myeloid and lymphoid neoplasms with FGFR1 rearrangements (*N* = 33). According to the central review committee, 24 (77.4%) patients had a CR with a cytogenetic complete response observed in 24 (75.8%) patients.^33^ An updated analysis reported that among patients with a CR, 81% and 47.6% had a >3-log and >2-log reduction in FGFR1 fusion transcripts by PCR, respectively.^34^ The most common treatment-related AEs were hyperphosphatemia (73%), alopecia (56%), and diarrhea (56%), and no patients had grade 3 or higher hyperphosphatemia.^35^

It could be hypothesized that pemigatinib’s efficacy in FGFR fusions and rearrangements stems from the driver mutational status of certain fusion partners compared with other FGF/FGFR alterations, which may be considered passenger mutations. Additionally, FGF/FGFR alterations specifically in mCRC may occur after exposure to VEGF-inhibitors such as bevacizumab (anti-VEGF antibody) and regorafenib (a multi-kinase inhibitor, including VEGFR1-3) as an escape mechanism for VEGF/VEGFR resistance to promote continued angiogenesis.^24-26,36^ FGF/FGFR alterations may be especially prominent in mCRC since VEGF inhibitors are recommended throughout the treatment course for mCRC patients without contraindications.^5,37-39^

Though the majority of patients included were RAS wildtype, it is unknown if downstream RAS or RAF mutations hinder the response to FGF/FGFR inhibition or develop as an escape mechanism after FGF/FGFR in inhibition, and further studies are needed to evaluate the effect RAS/RAF mutations may have on FGF/FGFR alterations. It is well known that patients with RAS mutations do not respond to EGFR inhibition (eg, cetuximab, panitumumab) since RAS acts as a downstream driver to promote proliferation.^40,41^

For example, Patient A had metastatic rectal adenocarcinoma and was initially treated with fluorouracil and oxaliplatin (FOLFOX)/bevacizumab (bev) followed by fluorouracil and irinotecan (FOLFIRI)/panitumumab. The patient then underwent molecular profiling which showed CCDN1 amplification, FGF19 amplification, FGF3 amplification, FGF4 amplification, APC mutation, and TP53 mutation. The patient was treated with pemigatinib and was able to be escalated to the higher dose for cycle 2, 18 mg daily, until progression of disease 2 months after initiation of pemigatinib. Patient B had metastatic left-sided colon adenocarcinoma initially treated with FOLFOX/bev and then second-line FOLFIRI/bev. Patient B underwent molecular profiling prior to initiation of therapy which showed KRAS G12S mutation, FLT3 amplification, CCND2 amplification, CDK8 amplification, APC splice variant, FGF23 amplification, SMAD4 loss, and TP53 mutation. In the third line, Patient B received targeted MEK plus CDK4/6 inhibitors (binimetinib and palbociclib) on a clinical trial prior to pemigatinib 13.5 mg daily for 1 month, requiring dose reduction to 9 mg daily until progression after 2 months. These two patients are representative of different tumor biology as patient A may have developed FGF alterations after exposure to VEGF inhibition, as an escape mechanism or passenger mutation, and tolerated pemigatinib at a higher dose. Patient B’s molecular profiling, done before exposure to therapy, had several other dominant mutations, including KRAS, which may have lessened response to pemigatinib. It is also possible that patient B developed other driver alterations over the course of therapy which were not tested prior to pemigatinib initiation. These two examples highlight the complexity of genomic alterations in tumors that can shift over time as patients are exposed to different therapies.

In both registrational studies for pemigatinib, the maximum dose given was 13.5 mg daily. In the FIGHT-202 trial, patients were treated with 13.5 mg daily for 2 weeks on, 1 week off, repeated in 21-day cycles. At this dose, 64% of patients experienced grade 3/4 AEs including hyperphosphatemia (12%), stomatitis (5%), hyponatremia (5%), abdominal pain (5%), and fatigue (5%).^32^ In the FIGHT-203 trial, pemigatinib was dosed at 13.5 mg daily continuously without dose escalation. At this dose, 73% of patients developed hyperphosphatemia with grade 3/4 AEs of stomatitis (17%) and anemia (15%).^35^ In our study, we found that 2 patients were able to be escalated to 18 mg daily which was both safe and tolerable but lacked clinical efficacy in FGFR/FGF-altered mCRC. This suggests that patients may be escalated to higher doses of pemigatinib if tolerating well, and whether this leads to higher clinical efficacy in FGFR fusion positive tumors could be explored.

## Conclusion

Tumor sequencing has led to the development and use of genomically targeted agents with the goal of prolonging OS in patients with metastatic cancer while minimizing off-target effects. In this population of patients with chemorefractory mCRC FGF/FGFR alterations (largely mutations and amplifications), targeting FGF/FGFR with pemigatinib did not lead to objective responses. Thus, driver genomic alterations may vary by tumor type leading to different outcomes with targeted therapy. Though pemigatinib monotherapy was not efficacious in this population, it was overall well-tolerated without any new safety signals. Continued exploration of targeted agents alone or in combination with cytotoxic agents is needed in this patient population with chemorefractory disease to improve survival and QOL.

## Supplementary Material

oyaf069_suppl_Supplementary_Material

## Data Availability

The data underlying this article will be shared on reasonable request to the corresponding author.
