# Supplementary material for: A phase II, multicenter, single-arm study of pemigatinib in patients with metastatic or unresectable colorectal cancer harboring FGFR alterations
Source: Oncologist. 2025 Jun 14;30(6):oyaf069. doi: 10.1093/oncolo/oyaf069 (PMC12166116; doi:10.1093/oncolo/oyaf069)
Supplement: oyaf069_suppl_Supplementary_Material [file oyaf069_suppl_supplementary_material.pdf]

Academic and Community Cancer Research United (ACCRU)

A Phase II, Multicenter, Single-Arm Study of Pemigatinib in Patients with Metastatic or Unresectable Colorectal Cancer Harboring FGFR Alterations

For any communications regarding this protocol, please contact the person indicated on the Protocol Resource page. This is a stand-alone document found on the ACCRU web site (www.ACCRU.org).

Study Chairs

ACCRU: Kristen K. Ciombor M.D.
Vanderbilt-Ingram Cancer Center
615-343-4669
615-343-7602 (FAX)
kristen.k.ciombor@vumc.org

FDA IND
Sponsor/Investigator: Tanios S. Bekaii-Saab, M.D. (Mayo Clinic)

Correlative Science Co-Chair: Andrew Nixon, Ph.D. (Duke Cancer Institute)

Statistician: Tyler Zemla √
507-293-2487

Drug Availability

Drug Company Supplied: Pemigatinib (IND144530)

√ Study contributor(s) not responsible for patient care.

Research Coordinating Center

Academic and Community Cancer Research United
200 First Street Southwest
Rochester, MN 55905
FAX# 507-538-0906

**Document History**	**(effective date)**
Activation ACCRU	July 15, 2019
Addendum 1	April 22, 2020
Addendum 2	December 09, 2020

Index

Schema
1.0 Background
2.0 Goals
3.0 Patient Eligibility
4.0 Test Schedule
5.0 Grouping Factors
6.0 Registration
7.0 Protocol Treatment
8.0 Dosage Modification Based on Adverse Events
9.0 Ancillary Treatment/Supportive Care
10.0 Adverse Event (AE) Reporting and Monitoring
11.0 Treatment Evaluation Using RECIST Guideline
12.0 Descriptive Factors
13.0 Treatment/Follow-up Decision at Evaluation of Patient
14.0 Body Fluid Biospecimens
15.0 Drug Information
16.0 Statistical Considerations and Methodology
17.0 Pathology Considerations/Tissue Biospecimens
18.0 Records and Data Collection Procedures
19.0 Budget
20.0 References
Appendix I – Acceptable Contraception
Appendix II – Examples of CYP3A4 Inducers and Inhibitors
Appendix III – Patient Medication Diary
Appendix IV – Patient Information Sheet
Appendix V - Linear Analogue Self Assessment (LASA)

Schema

Registration

↓

Treatment with Pemigatinib[2] until PD or a maximum of 35 cycles[1]

↓

Event Monitoring

Generic name: Pemigatinib
Availability: Incyte

PD at any time
Unacceptable adverse Events
Patient refusal
Investigator decision

↓

Event Monitoring

PD=Progressive Disease
[1]Cycle=21 Days
[2]Refer to section 7 for dosing Information

1.0 Background

1.1 Pemigatinib

Pemigatinib is an inhibitor of the fibroblast growth factor receptor (FGFR) family of receptor tyrosine kinases that has been investigated in the treatment of advanced malignancies. The FGFR pathway is critical for human development and plays a key role in cellular proliferation, migration, survival, and angiogenesis (Brooks, et al, 2012; Wesche, et al, 2011; Greulich, et al, 2011). Aberrant signaling through FGFR typically results from gene amplification or mutation, chromosomal translocation, and ligand-dependent activation of the receptors. Abrogation of this pathway leads to growth inhibition of tumor cells, and signaling of FGFRs has been identified as a possible escape mechanism for tumor angiogenesis when the vascular endothelial growth factor (VEGF) pathway is disrupted (Casanovas, et al, 2005; Berger, et al, 2008).

Activating alterations in the FGFR pathway are found in various malignancies (Marek, et al, 2009; Stransky, et al, 2014; Dienstmann, et al, 2014), including colorectal cancer (Goke, et al, 2013; Abdel-Rahman, 2015; Mathur, et al, 2014; Folprecht, et al, 2016), and inhibition of this pathway represents a therapeutic opportunity for patients with these genomically selected tumors. Refer to the Investigator's Brochure (IB) for additional background information on Pemigatinib , an oral inhibitor of FGFR1, FGFR2, and FGFR3.

1.2 Fibroblast Growth Factor Receptor Inhibitor in Oncology

The mammalian FGFR family is composed of 4 highly conserved receptors (FGFR1, FGFR2, FGFR3, and FGFR4) that have an extracellular ligand binding domain, a single transmembrane domain, and an intracellular tyrosine kinase domain. Eighteen FGF ligands, divided into canonical and hormonal FGFs, bind to FGFRs, leading to receptor dimerization, activation of the kinase domain, and transphosphorylation of the receptors (Eswarakumar, et al, 2005). Subsequent signal transduction occurs through phosphorylation of substrate proteins such as fibroblast growth factor receptor substrate 2, which leads to activation of the RAS–mitogen-activated protein kinase and PI3 kinase–protein kinase B pathways, and phospholipase Cγ, which activates the protein kinase C pathway. In some cellular contexts, signal transducer and activator of transcription proteins are also activated by FGFRs. Signaling through the FGF-FGFR pathway is tightly controlled through feedback regulation. Mitogen-activated protein kinase phosphatases and Sprouty proteins are upregulated upon FGFR stimulation and antagonize FGF-dependent activation of extracellular signal-regulated kinases. In many cases, FGFR pathway activation promotes cell proliferation, survival, and migration; however, cellular context plays an important role, and in certain tissues, FGFR signaling results in growth arrest and cellular differentiation (Dailey, et al, 2005).

There is strong genetic and functional evidence that dysregulation of FGFR can lead to the establishment and progression of cancer. Genetic alterations in FGFR1, FGFR2, and FGFR3 have been described in many tumor types (Knights and Cook 2010; Turner and Grose 2010). These include activating mutations, translocations, and gene amplification resulting in ligand-independent, constitutive activation of the receptors or aberrant ligand-dependent signaling through FGFRs.

Dysregulation of FGF ligands has also been reported in many human cancers. Preclinical

studies have shown that high levels of FGF ligands such as FGF2 promote cancer cell resistance to radiation, chemotherapeutics, and targeted cancer drugs (Fuks, et al, 1994; Pardo, et al, 2002; Terai, et al, 2013). In triple-negative breast cancer, upregulation of both FGF ligands and FGFR creates an autocrine loop that supports tumor cell growth and survival (Sharpe, et al, 2011). Clinically, detection of high levels of FGF2 in tumors is associated with poorer outcome in several tumor types, including NSCLC (Donnem, et al, 2009; Rades, et al, 2012).

A substantial body of evidence supports that a genetically activated FGFR pathway sensitizes FGFR-altered cancer cells to knockdown or inhibition of these receptors (Kunii, et al, 2008; Qing, et al, 2008; Weiss, et al, 2010; Lamont, et al, 2001). A large screen of more than 500 tumor cell lines with a selective FGFR inhibitor demonstrated that only a small percentage (5.9%) of all cells are sensitive to FGFR inhibition, and growth suppressed cell lines were highly enriched for FGFR alterations (Guagnano, et al, 2012). These results demonstrate that FGFR inhibitors are active in a targeted manger against cancers with activated FGFR pathways. An implication of these data is that selection based on molecular, genetic, or protein-based diagnostic tests for specific FGFR alterations in tumors is probably most important for identifying patients most likely to benefit from an FGFR inhibitor.

Results from early clinical studies of selective FGFR inhibitors other than Pemigatinib have also shown a tolerable safety profile for the class and have shown preliminary signs of clinical benefit in subjects selected for FGFR alterations. The safety and clinical activity of AZD4547 was evaluated in a Phase 1 study where subjects were prospectively selected for FGFR1 or FGFR2 gene amplification by fluorescent in situ hybridization (Kilgour, et al, 2014).

In the Phase 1 study of JNJ-42756493 in subjects with advanced malignancies, 65 subjects were treated on a once-daily (QD) continuous treatment schedule at 6 different increasing dose levels, plus 2 doses at 1-week-on/1-week-off dosing intervals (Tabernero, et al, 2015). Hyperphosphatemia (HP) was the leading dose-limiting toxicity resulting in treatment interruptions and discontinuations. Twenty-three subjects had FGFR1-4 or FGF3/FGF4 alterations. There were 4 confirmed responses and 1 unconfirmed partial response; 16 subjects had stable disease, eight of whom had stable disease for > 3 months.

A Phase 1 study of the selective pan-FGFR inhibitor BGJ398 has also shown a tolerable safety profile and preliminary efficacy in multiple tumor types (Sequist, et al, 2014). The study enrolled subjects with any type of FGFR genetic alteration. A 125 mg QD dose of BGJ398 was identified as the maximum tolerated dose. In the expansion cohort, 3 subjects achieved a confirmed PR and tumor shrinkage was observed in 2 other subjects that approached 30% (Nogova, et al, 2016). The disease control rate was 75%.

1.3 Metastatic Colorectal Cancer and FGFR inhibition

Colorectal cancer (CRC) is the third most common cancer in the United States in both men and women (Siegel, et al, 2018). Approximately 20% of patients present with metastatic disease at diagnosis, and a significant proportion of patients who are initially treated with potentially curative surgical resection experience disease recurrence and develop metastatic disease (Gill, et al, 2007). There are a number of standard intravenous and oral chemotherapies and biologic agents approved for the treatment of metastatic

CRC, but with time, resistance develops to these agents and patients with mCRC need additional therapeutic options.

Better characterization of the molecular variants that drive CRC progression have led to development of targeted therapies that improve clinical outcomes. For example, targeting the epidermal growth factor receptor (EGFR) with monoclonal antibodies (cetuximab, panitumumab) in patients with metastatic CRC (mCRC) whose tumors are *RAS* wildtype has improved patient survival. However, there remains an unmet need for additional genotype-driven therapeutic targets to treat mCRC patients.

Molecular alterations in FGFR has been previously described in CRC. Goke et al analyzed 454 primary tumors, and using a fluorescence in situ hybridization technique identified 24 (5.3%) samples that exhibited amplification in FGFR1 (Goke, et al, 2013). Mathur et al characterized a novel *FGFR2* amplification in the NCI-H716 CRC cell line that was derived from ascites of a poorly differentiated colon adenocarcinoma. These authors found that *FGFR2* gene amplification resulted in overexpression of the receptor at the cell surface and constitution activation (Mathur, et al, 2014). Most importantly, FGFR2 overexpression was required for NCI-H716 cell growth and survival *in vitro* and *in vivo*. Lastly, Folprecht et al analyzed 328 different cancer genes from 389 CRC patients, and observed 3.5% of samples exhibited FGFR 1/2/3 amplification (Folprecht, et al, 2016).

It is hypothesized that Pemigatinib , an oral inhibitor of FGFR1, FGFR2, and FGFR3, may have more activity in selected patients with metastatic colorectal cancer (mCRC) whose tumors harbor activating FGFR alterations. While this population is a small subset of patients with mCRC, given the need for better targeted therapies and the overall large prevalence of patients with mCRC, we propose a phase II, multicenter study of Pemigatinib in patients with mCRC harboring activating FGFR alterations.

1.4 Clinical Experience with Pemigatinib

Participants With Advanced Malignancies

As of the data cutoff date (25 NOV 2018), the safety and tolerability of pemigatinib administered alone or in combination with another chemotherapy/immunotherapy was being evaluated in 5 ongoing clinical studies in participants with advanced malignancies. Based on preliminary unaudited data from these ongoing studies, the most frequently occurring TEAEs (ie, incidence > 20%) were (in descending order of frequency) hyperphosphatemia, diarrhea, alopecia, fatigue, dry mouth, stomatitis, constipation, dysgeusia, nausea, decreased appetite, and anemia.
Additional details are presented by study in the following sections.

Study INCB 54828-101

Of the 150 participants with advanced malignancies who have been enrolled in Study INCB 54828-101 as of the data cutoff date, 43 participants have been administered pemigatinib in Part 1 (monotherapy dose escalation), 63 participants in Part 2 (monotherapy dose expansion), and 44 participants in Part 3 (pemigatinib combined with gemcitabine and cisplatin [8 participants], docetaxel [7 participants], pembrolizumab [23 participants], and trastuzumab [6 participants]).

Dose-limiting toxicities informed dose escalation procedures during Part 1 of the study. Among participants in Part 1, 28 received pemigatinib at doses of 1 to 20 mg QD on a 2 weeks-on/1-week-off therapy schedule (ie, interval dose regimen), and 15 participants received pemigatinib at doses of 9, 13.5, or 20 mg QD on a continuous schedule (ie, continuous dose regimen). There were no DLTs for the monotherapy dose regimens, and the monotherapy MTD was not reached. The recommended Part 2 dose for pemigatinib was determined to be 13.5 mg based on PD and clinical effect, and the recommended Phase 2 dose regimens for pemigatinib based on safety and PK data and preliminary signals of clinical benefit (data not shown) were 13.5 mg QD following the interval schedule and 13.5 mg QD following the continuous schedule.

Overall, 105 participants (99.1%) who received pemigatinib monotherapy (all doses and dose regimens combined) in Study INCB 54828-101 had TEAEs. Treatment-emergent AEs occurring in ≥ 10% of participants who received pemigatinib monotherapy (Parts 1 and 2 combined) are presented by dose and dose regimen and overall in Table 13. Consistent with the expected pharmacological effect of FGFR inhibition on serum phosphate levels, the most frequently occurring TEAE was hyperphosphatemia (74 participants [69.8%]; serum phosphate >5.5 mg/dL). Other frequent TEAEs (> 30%) included fatigue in 43 participants (40.6%), dry mouth in 39 participants (36.8%), and alopecia in 35 participants (33.0%). Comparison of the most frequently occurring TEAEs for the continuous and interval dose regimens suggests higher incidences of hyperphosphatemia (76.7% vs 67.1%), stomatitis (46.7% vs 22.4%), dry mouth

(43.3% vs 34.2%), diarrhea (43.3% vs 22.4%), constipation (40.0% vs 25.0%), alopecia (40.0% vs 30.3%), and nausea (33.3% vs 22.4%) with continuous dosing. Other TEAEs that occurred more frequently with continuous dosing included dry eye, pain in extremity, hypercalcemia, onycholysis, and paronychia.

Forty-five participants (42.5%) who received pemigatinib monotherapy had at least 1 SAE; the overall incidence of SAEs for the continuous dose regimen (56.7%) was higher than was seen for the interval dose regimen (36.8%). Pneumonia in 7 participants (6.6%) was the most frequently occurring SAE. Other SAEs occurring in more than 1 participant included back pain and disease progression in 4 participants (3.8%) each; abdominal pain, dehydration, fatigue, hyponatremia, and acute renal failure in 3 participants (2.8%) each; and blood bilirubin increased, cerebrovascular accident, constipation, hypotension, pain in extremity, pleural effusion, and pyrexia in 2 participants (1.9%) each. Within the eye disorders SOC, a single participant had an SAE of ocular hyperemia (Grade 2), which was considered unrelated to pemigatinib by the investigator.

A total of 11 participants (10.4%), 7 participants (9.2%) on an interval dose regimen and 4 participants (13.3%) on a continuous dose regimen, had SAEs with a fatal outcome: disease progression in 4 participants (3.8%) and

pneumonia, malignant neoplasm progression (ie, disease progression), cerebrovascular accident, intracranial hemorrhage, multiorgan failure, esophageal varices hemorrhage, pneumonia, respiratory failure, and acute respiratory failure secondary to acute anemia (verbatim term) in 1 participant (0.9%) each. None of these fatal events were assessed as related to pemigatinib by the investigator.

Eleven participants (10.4%) discontinued pemigatinib monotherapy due to TEAEs; pneumonia in 3 participants (2.8%) and dehydration and small intestinal obstruction in 2 participants (1.9%) each were the only TEAEs leading to discontinuation of pemigatinib that occurred in more than 1 participant.

Table 13: Summary of Treatment-Emergent Adverse Events Occurring in ≥ 10% of Participants on Pemigatinib Monotherapy in Study INCB 54828-101 (Parts 1 and 2 Combined) in Decreasing Order of Frequency

	Pemigatinib Interval Dose Regimen[a]						Pemigatinib Continuous Dose Regimen[b]				
MedDRA Preferred Term, n (%)	1/2/4 mg (N = 3)	6 mg (N = 4)	9 mg (N = 13)	13.5 mg (N = 50)	20 mg (N = 6)	Subtotal (N = 76)	9 mg (N = 8)	13.5 mg (N = 16)	20 mg (N = 6)	Subtotal (N = 30)	Total (N = 106)
Hyperphosphataemia	0	1 (25.0)	8 (61.5)	38 (76.0)	4 (66.7)	51 (67.1)	4 (50.0)	14 (87.5)	5 (83.3)	23 (76.7)	74 (69.8)
Fatigue	1 (33.3)	1 (25.0)	7 (53.8)	19 (38.0)	2 (33.3)	30 (39.5)	5 (62.5)	5 (31.3)	3 (50.0)	13 (43.3)	43 (40.6)
Dry mouth	0	1 (25.0)	6 (46.2)	17 (34.0)	2 (33.3)	26 (34.2)	2 (25.0)	7 (43.8)	4 (66.7)	13 (43.3)	39 (36.8)
Alopecia	0	0	7 (53.8)	15 (30.0)	1 (16.7)	23 (30.3)	2 (25.0)	7 (43.8)	3 (50.0)	12 (40.0)	35 (33.0)
Constipation	0	0	3 (23.1)	14 (28.0)	2 (33.3)	19 (25.0)	3 (37.5)	5 (31.3)	4 (66.7)	12 (40.0)	31 (29.2)
Stomatitis	0	0	3 (23.1)	11 (22.0)	3 (50.0)	17 (22.4)	4 (50.0)	7 (43.8)	3 (50.0)	14 (46.7)	31 (29.2)
Diarrhoea	0	1 (25.0)	2 (15.4)	12 (24.0)	2 (33.3)	17 (22.4)	1 (12.5)	8 (50.0)	4 (66.7)	13 (43.3)	30 (28.3)
Nausea	1 (33.3)	1 (25.0)	5 (38.5)	8 (16.0)	2 (33.3)	17 (22.4)	2 (25.0)	7 (43.8)	1 (16.7)	10 (33.3)	27 (25.5)
Decreased appetite	0	2 (50.0)	2 (15.4)	12 (24.0)	1 (16.7)	17 (22.4)	0	4 (25.0)	2 (33.3)	6 (20.0)	23 (21.7)
Dysgeusia	1 (33.3)	0	5 (38.5)	8 (16.0)	2 (33.3)	16 (21.1)	1 (12.5)	3 (18.8)	3 (50.0)	7 (23.3)	23 (21.7)
Anaemia	1 (33.3)	0	3 (23.1)	10 (20.0)	2 (33.3)	16 (21.1)	3 (37.5)	2 (12.5)	1 (16.7)	6 (20.0)	22 (20.8)
Abdominal pain	0	0	3 (23.1)	11 (22.0)	1 (16.7)	15 (19.7)	1 (12.5)	4 (25.0)	1 (16.7)	6 (20.0)	21 (19.8)
Vomiting	1 (33.3)	0	4 (30.8)	8 (16.0)	1 (16.7)	14 (18.4)	1 (12.5)	2 (12.5)	2 (33.3)	5 (16.7)	19 (17.9)
Aspartate aminotransferase increased	1 (33.3)	0	4 (30.8)	7 (14.0)	0	12 (15.8)	2 (25.0)	4 (25.0)	0	6 (20.0)	18 (17.0)
Hypophosphataemia	0	0	2 (15.4)	10 (20.0)	1 (16.7)	13 (17.1)	0	4 (25.0)	1 (16.7)	5 (16.7)	18 (17.0)
Dehydration	1 (33.3)	1 (25.0)	0	7 (14.0)	1 (16.7)	10 (13.2)	2 (25.0)	2 (12.5)	1 (16.7)	5 (16.7)	15 (14.2)
Dry eye	0	0	1 (7.7)	7 (14.0)	1 (16.7)	9 (11.8)	0	5 (31.3)	1 (16.7)	6 (20.0)	15 (14.2)
Pain in extremity	0	1 (25.0)	3 (23.1)	3 (6.0)	1 (16.7)	8 (10.5)	1 (12.5)	5 (31.3)	1 (16.7)	7 (23.3)	15 (14.2)
Alanine aminotransferase increased	1 (33.3)	0	4 (30.8)	4 (8.0)	0	9 (11.8)	2 (25.0)	3 (18.8)	0	5 (16.7)	14 (13.2)
Cough	1 (33.3)	0	1 (7.7)	6 (12.0)	2 (33.3)	10 (13.2)	1 (12.5)	3 (18.8)	0	4 (13.3)	14 (13.2)
Vision blurred	1 (33.3)	1 (25.0)	2 (15.4)	4 (8.0)	2 (33.3)	10 (13.2)	0	3 (18.8)	1 (16.7)	4 (13.3)	14 (13.2)
Weight decreased	2 (66.7)	0	2 (15.4)	5 (10.0)	2 (33.3)	11 (14.5)	1 (12.5)	0	2 (33.3)	3 (10.0)	14 (13.2)
Blood alkaline phosphatase increased	1 (33.3)	0	3 (23.1)	6 (12.0)	0	10 (13.2)	2 (25.0)	1 (6.3)	0	3 (10.0)	13 (12.3)
Hypercalcaemia	1 (33.3)	0	1 (7.7)	5 (10.0)	0	7 (9.2)	2 (25.0)	3 (18.8)	1 (16.7)	6 (20.0)	13 (12.3)
Back pain	1 (33.3)	0	4 (30.8)	4 (8.0)	0	9 (11.8)	1 (12.5)	2 (12.5)	0	3 (10.0)	12 (11.3)
Onycholysis	0	0	1 (7.7)	5 (10.0)	0	6 (7.9)	1 (12.5)	5 (31.3)	0	6 (20.0)	12 (11.3)
Paronychia	0	0	1 (7.7)	2 (4.0)	2 (33.3)	5 (6.6)	1 (12.5)	4 (25.0)	2 (33.3)	7 (23.3)	12 (11.3)
Arthralgia	0	1 (25.0)	3 (23.1)	2 (4.0)	2 (33.3)	8 (10.5)	0	2 (12.5)	1 (16.7)	3 (10.0)	11 (10.4)
Hyponatraemia	0	0	1 (7.7)	5 (10.0)	1 (16.7)	7 (9.2)	0	3 (18.8)	1 (16.7)	4 (13.3)	11 (10.4)
Nail discolouration	0	0	2 (15.4)	5 (10.0)	0	7 (9.2)	1 (12.5)	2 (12.5)	1 (16.7)	4 (13.3)	11 (10.4)

Note: Participants were counted once under each MedDRA preferred term. Adverse events are ordered by the descending frequency in the total column.
Note: Treatment-emergent AEs are any AEs either reported for the first time or worsening of a pre-existing event after first dose of study drug.
[a] Pemigatinib was administered QD on a 2-weeks-on/1-week-off therapy schedule.
[b] Pemigatinib was administered QD.

Treatment-emergent AEs occurring in ≥ 10% of participants on combination therapy in Study INCB 54828-101 (Part 3) are presented by treatment group and overall in Table 14. The most frequently occurring TEAEs for each treatment combination were as follows:

- **Pemigatinib + Gemcitabine + Cisplatin:** Anemia (8/8 participants); blood creatinine increased, constipation, fatigue, and nausea (5/8 participants each); and ALT increased, AST increased, diarrhea, hyperphosphatemia, hyponatremia,

thrombocytopenia, and WBC count decreased (4/8 participants each).

- **Pemigatinib + Docetaxel:** Hyperphosphatemia, diarrhea, and dysgeusia (6/7 participants each); fatigue and dehydration (5/7 participants each); and nausea (4/7 participants).

- **Pemigatinib + Pembrolizumab:** Hyperphosphatemia (17/23 participants), anemia (11/23 participants), and diarrhea and decreased appetite (10/23 participants each)

- **Pemigatinib + Trastuzumab:** Alopecia, cough, and hyperphosphatemia (4/6 participants each) and decreased appetite, diarrhea, and dry mouth (3/6 participants each).

A single participant who received pemigatinib + docetaxel had DLTs of fatigue (Grade 3) anddehydration (Grade 3). No other participants on combination therapy had a DLT.

A total of 18 participants (40.9%) on combination therapy had at least 1 SAE: 4/8 participants on pemigatinib + gemcitabine + cisplatin, 6/7 participants on pemigatinib + docetaxel, and 8/23 participants on pemigatinib + pembrolizumab. The most frequently occurring SAEs across all combination therapy cohorts were dehydration in 3 participants (6.8%) and anemia and acute renal failure in 2 participants (4.5%) each. By treatment combination, the only SAE occurring in more than 1 participant was dehydration in 2 participants in the pemigatinib + docetaxel cohort. Two participants (4.5%) had SAEs with a fatal outcome: completed suicide (pembrolizumab +pemigatinib 9 mg) and disease progression (gemcitabine + cisplatin + pemigatinib 13.5 mg).Neither of these events was assessed as related to pemigatinib.

Five participants (11.4%) on combination therapy had TEAEs leading to discontinuation of pemigatinib. No event leading to discontinuation occurred in more than 1 participant.

Table 14: Summary of Treatment-Emergent Adverse Events Occurring in ≥ 10% of Participants on Pemigatinib Combination Therapy in Study INCB 54828-101 (Part 3) in Decreasing Order of Frequency

	Pemigatinib + GEM + CIS		Pemigatinib + DOC	Pemigatinib + PEM				Pemigatinib + TRAS	
	Interval Dose Regimen[a]		Interval Dose Regimen[a]	Interval Dose Regimen[a]			Continuous Dose Regimen[b]	Interval Dose Regimen[a]	
MedDRA Preferred Term, n (%)	9 mg (N = 1)	13.5 mg (N = 7)	13.5 mg (N = 7)	9 mg (N = 3)	13.5 mg (N = 14)	Subtotal (N = 17)	13.5 mg (N = 6)	13.5 mg (N = 6)	Total (N = 44)
Hyperphosphataemia	1 (100.0)	3 (42.9)	6 (85.7)	3 (100.0)	11 (78.6)	14 (82.4)	3 (50.0)	4 (66.7)	31 (70.5)
Anaemia	1 (100.0)	7 (100.0)	3 (42.9)	1 (33.3)	8 (57.1)	9 (52.9)	2 (33.3)	2 (33.3)	24 (54.5)
Diarrhoea	0	4 (57.1)	6 (85.7)	1 (33.3)	7 (50.0)	8 (47.1)	2 (33.3)	3 (50.0)	23 (52.3)
Fatigue	0	5 (71.4)	5 (71.4)	1 (33.3)	5 (35.7)	6 (35.3)	1 (16.7)	2 (33.3)	19 (43.2)
Alopecia	0	1 (14.3)	3 (42.9)	0	6 (42.9)	6 (35.3)	2 (33.3)	4 (66.7)	16 (36.4)
Constipation	1 (100.0)	4 (57.1)	3 (42.9)	0	5 (35.7)	5 (29.4)	1 (16.7)	2 (33.3)	16 (36.4)
Decreased appetite	0	2 (28.6)	1 (14.3)	0	9 (64.3)	9 (52.9)	1 (16.7)	3 (50.0)	16 (36.4)
Dry mouth	1 (100.0)	2 (28.6)	1 (14.3)	0	4 (28.6)	4 (23.5)	4 (66.7)	3 (50.0)	15 (34.1)
Dysgeusia	0	2 (28.6)	6 (85.7)	0	3 (21.4)	3 (17.6)	3 (50.0)	1 (16.7)	15 (34.1)
Nausea	1 (100.0)	4 (57.1)	4 (57.1)	0	3 (21.4)	3 (17.6)	1 (16.7)	2 (33.3)	15 (34.1)
Stomatitis	1 (100.0)	2 (28.6)	2 (28.6)	1 (33.3)	5 (35.7)	6 (35.3)	1 (16.7)	2 (33.3)	14 (31.8)
Blood creatinine increased	1 (100.0)	4 (57.1)	1 (14.3)	1 (33.3)	4 (28.6)	5 (29.4)	0	1 (16.7)	12 (27.3)
Dehydration	1 (100.0)	2 (28.6)	5 (71.4)	1 (33.3)	2 (14.3)	3 (17.6)	1 (16.7)	0	12 (27.3)
Aspartate aminotransferase increased	0	4 (57.1)	0	0	5 (35.7)	5 (29.4)	1 (16.7)	1 (16.7)	11 (25.0)
Cough	0	1 (14.3)	1 (14.3)	0	5 (35.7)	5 (29.4)	0	4 (66.7)	11 (25.0)
Alanine aminotransferase increased	0	4 (57.1)	0	0	5 (35.7)	5 (29.4)	1 (16.7)	0	10 (22.7)
Dyspnoea	0	2 (28.6)	0	1 (33.3)	4 (28.6)	5 (29.4)	1 (16.7)	2 (33.3)	10 (22.7)
Vomiting	0	3 (42.9)	3 (42.9)	1 (33.3)	2 (14.3)	3 (17.6)	1 (16.7)	0	10 (22.7)
Dry eye	0	0	1 (14.3)	0	4 (28.6)	4 (23.5)	2 (33.3)	2 (33.3)	9 (20.5)
Hyponatraemia	1 (100.0)	3 (42.9)	2 (28.6)	1 (33.3)	2 (14.3)	3 (17.6)	0	0	9 (20.5)
Hypomagnesaemia	1 (100.0)	1 (14.3)	1 (14.3)	1 (33.3)	4 (28.6)	5 (29.4)	0	0	8 (18.2)
Abdominal pain	1 (100.0)	1 (14.3)	1 (14.3)	0	2 (14.3)	2 (11.8)	2 (33.3)	0	7 (15.9)
Back pain	0	0	2 (28.6)	1 (33.3)	2 (14.3)	3 (17.6)	1 (16.7)	1 (16.7)	7 (15.9)
Blood alkaline phosphatase increased	0	1 (14.3)	1 (14.3)	1 (33.3)	2 (14.3)	3 (17.6)	1 (16.7)	1 (16.7)	7 (15.9)
Hypercalcaemia	0	1 (14.3)	1 (14.3)	1 (33.3)	4 (28.6)	5 (29.4)	0	0	7 (15.9)

Table 14: Summary of Treatment-Emergent Adverse Events Occurring in ≥ 10% of Participants on Pemigatinib Combination Therapy in Study INCB 54828-101 (Part 3) in Decreasing Order of Frequency (Continued)

	Pemigatinib + GEM + CIS		Pemigatinib + DOC	Pemigatinib + PEM				Pemigatinib + TRAS	
	Interval Dose Regimen[a]		Interval Dose Regimen[a]	Interval Dose Regimen[a]			Continuous Dose Regimen[c]	Interval Dose Regimen[a]	
MedDRA Preferred Term, n (%)	9 mg (N = 1)	13.5 mg (N = 7)	13.5 mg (N = 7)	9 mg (N = 3)	13.5 mg (N = 14)	Subtotal (N = 17)	13.5 mg (N = 6)	13.5 mg (N = 6)	Total (N = 44)
Hypoalbuminaemia	1 (100.0)	0	1 (14.3)	1 (33.3)	2 (14.3)	3 (17.6)	0	1 (16.7)	6 (13.6)
Hypokalaemia	1 (100.0)	2 (28.6)	0	0	3 (21.4)	3 (17.6)	0	0	6 (13.6)
Hypotension	0	0	2 (28.6)	1 (33.3)	3 (21.4)	4 (23.5)	0	0	6 (13.6)
Neutropenia	0	3 (42.9)	3 (42.9)	0	0	0	0	0	6 (13.6)
Pyrexia	1 (100.0)	0	0	0	1 (7.1)	1 (5.9)	2 (33.3)	2 (33.3)	6 (13.6)
Vision blurred	0	1 (14.3)	1 (14.3)	0	3 (21.4)	3 (17.6)	0	1 (16.7)	6 (13.6)
Dizziness	0	3 (42.9)	0	0	2 (14.3)	2 (11.8)	0	0	5 (11.4)
Headache	0	1 (14.3)	1 (14.3)	0	1 (7.1)	1 (5.9)	0	2 (33.3)	5 (11.4)
Hyperglycaemia	0	1 (14.3)	1 (14.3)	1 (33.3)	2 (14.3)	3 (17.6)	0	0	5 (11.4)
Oedema peripheral	0	1 (14.3)	2 (28.6)	0	1 (7.1)	1 (5.9)	1 (16.7)	0	5 (11.4)
Paronychia	0	0	2 (28.6)	0	2 (14.3)	2 (11.8)	0	1 (16.7)	5 (11.4)
White blood cell count decreased	1 (100.0)	3 (42.9)	0	0	1 (7.1)	1 (5.9)	0	0	5 (11.4)
Weight decreased	0	1 (14.3)	3 (42.9)	0	0	0	1 (16.7)	0	5 (11.4)

CIS = cisplatin; DOC = docetaxel; GEM = gemcitabine; PEM = pembrolizumab; TRAS = trastuzumab.

Note: Participants were counted once under each MedDRA preferred term. Adverse events are ordered by the descending frequency in total column.

Note: Treatment-emergent AEs are any AEs either reported for the first time or worsening of a pre-existing event after first dose of study drug.

a Pemigatinib was administered QD on a 2-weeks-on/1-week-off therapy schedule.

b Pemigatinib was administered QD.

1.5 Justification for Dose and Regimen

Pemigatinib will be administered at 13.5 mg QD, continuously, for a cycle. Each cycle is 3 weeks. This dose and dosing schedule was selected based on emerging clinical and safety data from the INCB 54828-101 study, where the continuous dosing regimen has been tested in 12 and 14 participants at 9 mg and 13.5 mg QD continuous administration, respectively. In Study INCB 54828-101, the emerging safety data demonstrated that tolerability of the continuous dosing regimen of pemigatinib was comparable to that of intermittent dosing.

The hypothesis with a targeted therapy is that continued inhibition of the aberrant receptor may increase the potential for benefit from the treatment. Therefore, administering pemigatinib continuously will allow that consistent inhibition of the aberrant FGFR receptor in this population. Continuous dosing would still allow for dose holds for safety reasons with criteria and procedures for dose interruptions and adjustments clearly outlined in the Protocol.

Hyperphosphatemia is an expected on-target pharmacological effect of FGFR inhibition. The incidence of hyperphosphatemia, defined as any post-baseline phosphate level exceeding 5.5 mg/dL, has been observed in the majority of study participants treated with pemigatinib (refer to the IB for complete data). Some participants do not achieve hyperphosphatemia, and it is estimated that pharmacological concentration of pemigatinib in these participants is lower (see Figure 2). Therefore, up titration of pemigatinib will be used to increase the exposure of pemigatinib in participants who do not achieve hyperphosphatemia when treated with 13.5 mg QD. The goal is to increase the serum concentration of pemigatinib.

The increase in serum phosphorus observed after treatment with pemigatinib was exposure-dependent and followed a sigmoid relationship. A population Emax model of pemigatinib AUC and maximal serum phosphate change from baseline was developed. For those participants treated with pemigatinib 13.5 mg who did not develop hyperphosphatemia, AUC for pemigatinib 18 mg was estimated using a linear exposure relationship. Maximal serum phosphate change from baseline for each individual was then estimated using a population model. The maximal serum phosphate after treatment with pemigatinib 18 mg was calculated by adding the baseline of serum phosphate. The simulation suggested that the serum phosphate would increase above 5.5 mg/dL after treatment with pemigatinib 18 mg for the participants treated with pemigatinib 13.5 mg who did not develop hyperphosphatemia.

Figure 2: Comparison of Steady State Exposures for Pemigatinib 13.5 mg QD Between Subjects With Nonhyperphosphatemia and Hyperphosphatemia

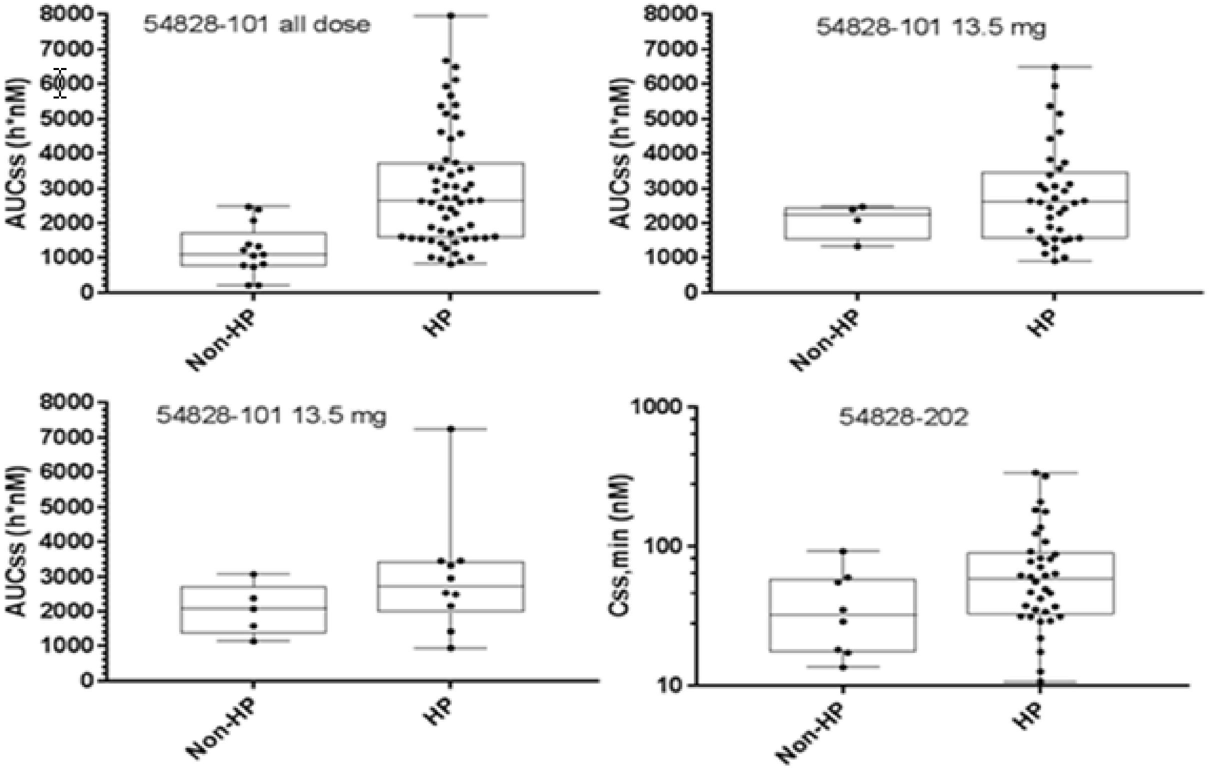

AUCss = area under the curve at steady state; Css, min = minimum blood plasma concentration at steady state; HP = hyperphosphatemia.

Any participant who does not reach the target serum phosphate level of > 5.5 mg/dL at any time during Cycle 1, and who is compliant with taking study drug and does not experience an ongoing Grade 2 or higher treatment-related AEs, will increase the daily dose to 18 mg starting at Cycle 2 Day 1. Participants who are titrated up to 18 mg QD will begin the next cycle at the new dose level and must agree to all Cycle 1 assessments (PK and safety assessments [hematology and blood chemistry]). Up-titration may occur no earlier than on Day 1 of Cycle 2, so that participants are observed for phosphate level and AEs at least for 1 cycle.
For participants who are up-titrated from 13.5 mg to 18 mg, a maximum of 2 dose level reductions is also recommended: 18 mg can be decreased to 13.5 mg, and if additional dose reduction is required, participants can decrease to 9 mg. Dose reductions below 9 mg are not allowed."

A CYP3A4-mediated drug-drug interaction study (INCB 54828-104) indicated there is evidence of a clinically significant effect on pemigatinib exposure when co-administered with a potent CYP3A4 inhibitor, itraconazole (increased pemigatinib AUC by 88%) or

potent CYP3A4 inducer, rifampin (decrease pemigatinib AUC by 85%). A PBPK model was developed and validated using in vitro and clinical DDI data. PBPK model-simulated pemigatinib AUCs were increased by approximately 50% for moderate CYP3A4 inhibitors and decreased by more than 50% for moderate CYP3A4 inducers. In addition, PBPK modeling shows no DDI effect when pemigatinib was coadministered with a weak CYP3A4 inhibitor or inducer.

1.6 Pharmacokinetic/Pharmacodynamic Summary

Pemigatinib exhibited linear pharmacokinetics (PK) over the dose range evaluated, with rapid oral absorption and a biphasic elimination, with a terminal half-life range of 10.9 to 31.4 hours. The projected average inhibition of FGFR2 based on PK and *in vitro* potency of Pemigatinib ranged from 41% at 1 mg to 97% at 20mg. Consistent with this projection, the observed inhibition of pFGFR2 in KATOIII cells spiked to *ex vivo* whole blood samples collected from subjects at trough was 82% after the 13.5 mg QD dose and 64% after the 9 mg QD dose. The steady-state plasma concentration of Pemigatinib after 13.5 mg QD dose that exceeded *in vivo* IC50 over a 24-hour dosing period is shown in Figure 1.

Figure 11: Pemigatinib Plasma Concentrations (Mean ± SE) at Steady State After 13.5 mg QD Oral Doses of Pemigatinib (Studies INCB 54828-101 Parts 1 and 2, N = 53 and INCB 54828-102, N = 17)

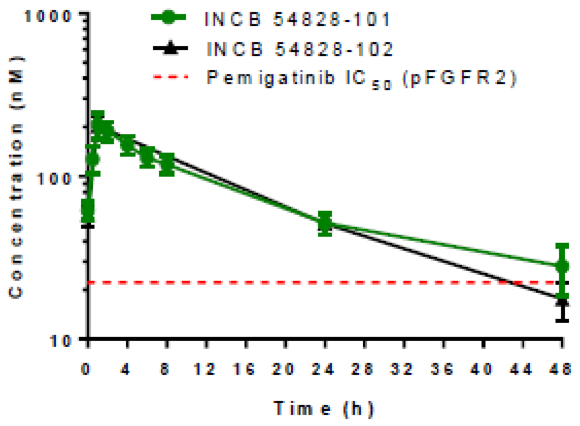

The magnitude and frequency of hyperphosphatemia was also dose dependent. In the 9 mg cohorts of Part 1 and Part 2, 4 of the 6 patients experienced hyperphosphatemia. In the 13.5 mg cohort of Part 1, all 6 subjects developed hyperphosphatemia, which was managed with a low-phosphate diet and introduction of phosphate binders. Further, the increase in serum phosphorus observed after treatment with Pemigatinib was exposure-dependent (see Figure 2).

Figure 2: Serum Phosphate Versus Exposure

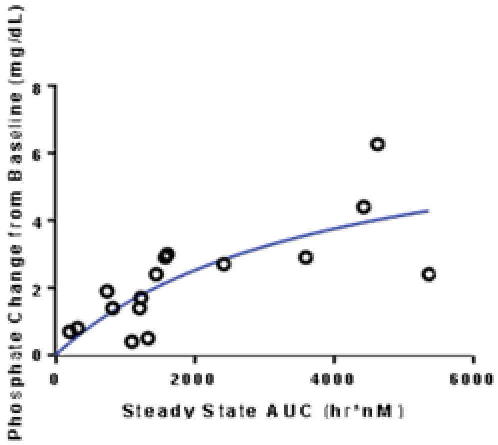

Therefore, based on a manageable safety profile and favorable PK/PD profile, the dose for this Phase 2 study is 13.5 mg.

1.7 Phototoxicity

There are no preclinical data available to date on the potential phototoxicity of Pemigatinib. Therefore, subjects enrolled in this study will be instructed by the site staff to take precaution to protect themselves from the sun/ultraviolet light. This includes wearing long sleeves, long trousers, hats, and sunglasses.

1.8 Correlative Research

1.81 Peripheral blood samples

Advances in next-generation sequencing (NGS) technology have allowed for the opportunity to better understand the relationship between tumor biology and the genomic landscape. However, serial sampling of tumor tissues is challenging in most tumor types given the anatomic location and requirement of adequate quantity of tumor cells among a background of normal cells and stroma. Thus, the emergence of the “liquid biopsy” and advances in sequencing technology allow for the opportunity to serially monitor the dynamics of specific tumor mutations (Amodovar, et al, 2018; Montagut, et al, 2018; Strickler, et al, 2018). Diaz and colleagues were one of the first groups to show that *KRAS* mutations could be detected in the sera of CRC patients who, before receiving monotherapy with panitumumab, were *KRAS* wildtype (Diaz, et al, 2012). Strickler *et al.* performed large-scale genomic profiling of cell-free DNA (cfDNA) from patients with CRC and observed genomic alterations at frequencies comparable to databases with large-scale tumor sequencing data (Strickler, et al, 2018) (i.e., The Cancer Genome Atlas and Genomics Evidence Neoplasia Information Exchange). Specifically, these authors reported the ability to detect, with adequate sensitivity, *RAS* mutations and mutations in the extracellular domain of

EGFR in cfDNA. A recent clinical trial evaluating a novel EGFR mAb (Sym004) in *RAS* wildtype CRC patients that have progressed on panitumumab or cetuximab analyzed circulating tumor DNA throughout treatment and observed the emergence of extremely heterogeneous molecular landscapes where subclones with distinct resistance mechanisms coexist in one patient (Montagut, et al, 2018). Another recent clinical trial observed the emergence of *KRAS* and *NRAS* mutations upon disease progression in *BRAF*-mutant CRC patients who were receiving a triplet drug combination designed to inhibit BRAF, EGFR, and MEK (Corcoran, et al, 2018). Thus, analyzing cfDNA is becoming a viable clinical technique to serially monitor the emergence of resistance mechanisms that are driving the observed disease progression in CRC.

Serial blood samples will be collected throughout the study according to the test table to analyze dynamic changes in cfDNA as a function of treatment. The objective is to explore the potential mutational patterns that could be associated with tumor response and disease progression. Samples will be collected from all patients. Any detected single nucleotide variants, insertions and deletions of any length, chimeric gene fusions that arise through chromosomal rearrangement, and copy number gain or copy number loss will be recorded longitudinally for each patient.

1.82 Archival tissue

Archival tissue, if available, will be collected from each patient. Molecular profiling will be performed on the available tissue. Concordance results between tissue and the subsequent liquid biopsy results will be assessed.

2.0 Goals

2.1 Primary

2.11 To assess overall response rate (ORR) of Pemigatinib in patients with metastatic or unresectable colorectal cancer harboring activating FGFR alterations.

2.2 Secondary

2.21 To assess the clinical benefit rate (complete response + partial response + stable disease) with Pemigatinib .

2.22 To assess progression free survival (PFS) and overall survival (OS) with Pemigatinib.

2.23 Assess changes in patient quality of life (QOL) as measured by the linear analogue self-assessment (LASA) questionnaire.

2.24 Assess the frequency and severity of adverse events.

2.3 Correlative Research

2.31 To assess plasma pharmacodynamic biomarkers of response and resistance to

therapy.

2.32 To explore any correlation between tissue and blood based biomarkers and clinical outcomes.

3.0 Patient Eligibility

NOTE: Waivers to eligibility criteria are not allowed per ACCRU policy

3.1 Registration – Inclusion Criteria

3.11 Histologically or cytologically confirmed diagnosis of metastatic or unresectable colorectal cancer (mCRC), based on documentation from local or outside review of pathology according to each site's established institutional procedure

3.12 Documentation of an activating genomic alteration(s) in FGF/FGFR1-3 (gain of function mutations, translocations, and amplifications allowed) in tumor tissue or blood tested at a Clinical Laboratory Improvement Amendments (CLIA) – certified laboratory.

3.13 Provide informed written consent.

3.14 Age ≥ 18 years.

3.15 Patient must have received and progressed on, or be intolerant to, each of the following treatments for mCRC (or have contraindication to these treatments):

- Fluoropyrimidine.
- Oxaliplatin.
- Irinotecan.
- Pembrolizumab
- Nivolumab
- Anti-VEGF (vascular endothelial growth factor) monoclonal antibody, if eligible for this therapy.
- Anti-EGFR (epidermal growth factor receptor) monoclonal antibody, if eligible for this therapy.

3.16 Measurable disease as defined in Section 11.0

3.17 ECOG Performance Status (PS) of 0, 1, or 2. Form is available on the ACCRU web site.

3.18 The following laboratory values obtained ≤ 28 days prior to registration:

- Absolute neutrophil count (ANC) ≥ 1500/mm^3.

- Platelet count ≥ 100,000/mm^3.
- Hemoglobin ≥ 9.0 g/dL.
- Total bilirubin ≤ 1.5x upper limit of normal (ULN), or ≤ 2.5x ULN if patient has Gilbert syndrome or disease involving the liver.
- Aspartate aminotransferase (AST) and alanine aminotransferase (ALT) ≤ 2.5x ULN (or ≤ 5x ULN in presence of suspected liver metastases).
- Serum phosphate < institutional ULN.
- Serum calcium within institutional normal range, or
  serum albumin-corrected calcium within institutional normal range (if serum albumin is outside of the institutional normal range).
- Potassium levels > institutional lower limit of normal (supplementation can be used to correct potassium level during screening).
- Serum creatinine ≤ 1.5x ULN, or
  calculated creatinine clearance > 30 mL/min using the Cockcroft-Gault formula (see below) or 24-hours urine collection analysis:

Cockcroft-Gault Equation:

$$\text{Creatinine clearance for males} = \frac{(140 - \text{age})(\text{weight in kg})}{(72)(\text{serum creatinine in mg/dL})}$$

$$\text{Creatinine clearance for females} = \frac{(140 - \text{age})(\text{weight in kg})(0.85)}{(72)(\text{serum creatinine in mg/dL})}$$

3.19 Corrected QT interval (QTc) by Fridericia's method (QTcF) assessed by electrocardiogram (ECG) completed ≤ 28 days prior to registration, and resulted as:

- QTcF ≤ 450 msec in men, or
- QTcF ≤ 470 msec in women.

3.19a Negative serum pregnancy test completed ≤ 7 days prior to registration, for women of childbearing potential only. See Appendix I for definition of WOCBP.

3.19b Willing to provide tissue and blood samples for correlative research purposes (see Sections 6.0, 14.0 and 17.0).

3.19c Willing to allow transfer of tissue and blood samples, clinical information, and outcome data collected from this trial for future research (see Sections 6, 14, and 17).

3.2 Registration – Exclusion Criteria

3.21 Prior treatment with Pemigatinib.

3.22 Prior treatment with a selective FGFR inhibitor ≤ 180 days (6 months) prior to registration.

3.23 Known hypersensitivity or severe reaction to an FGFR inhibitor, or to the excipients of Pemigatinib (i.e. microcrystalline cellulose, sodium starch glycolate, and magnesium stearate).

3.24 Current evidence of clinically significant corneal or retinal disorder confirmed by ophthalmologic examination.

3.25 Treatment with other investigational study drug for any indication for any reason, or receipt of anticancer medications ≤ 14 days prior to registration.

3.26 Major surgery ≤ 28 days prior to registration.

3.27 External beam radiation therapy ≤ 28 days prior to registration, or palliative radiation for non-CNS disease ≤ 14 days prior to registration.

3.28 Brain metastases, central nervous system (CNS) metastases, leptomeningeal disease, or spinal cord compression

NOTE: Patients who are asymptomatic or previously treated and stable, without evidence of progression for ≥ 28 days prior to registration are eligible

NOTE: Patients taking concomitant corticosteroids and/or anticonvulsants are allowed if patient is on a stable or decreasing dose of such treatment for ≥ 28 days prior to registration.

3.29 History or presence of significant cardiovascular disease or condition including:

- Uncontrolled angina pectoris (Canadian Cardiovascular Society Grade II-IV despite medical therapy).
- Congestive heart failure (New York Heart Association Class III or IV).
- Uncontrolled arrhythmia requiring therapy. Note: Patients with a pacemaker and well-controlled rhythm for ≥ 28 days prior to registration are not excluded.
- Any of the following occurring ≤ 6 months prior to registration: Myocardial infarction, angioplasty, cardiac stenting, coronary/peripheral artery bypass graft, cerebrovascular accident or transient ischemic attack.

3.29a Failure to adequately recover (i.e. to ≤ Grade 1(according to CTCAE v.5) or to pre-treatment baseline) from adverse events (AEs) deemed by the investigator as clinically significant and attributed to prior therapy.
Exception: alopecia.

3.29b Current use of prohibited medication as described in Section 9.11.

3.29c Use of any potent CYP3A4 inhibitors or inducers or moderate CYP3A4 inducers (see Appendix II) ≤14 days or 5 half-lives (whichever is shorter) prior to registration. Note: Topical ketoconazole will be allowed.

3.29d History of hypovitaminosis D requiring supraphysiologic doses to replenish the deficiency. Note: Patients receiving vitamin D food supplements are allowed.

3.29e History and/or current evidence of ectopic mineralization/calcification, including but not limited to soft tissue, kidneys, intestine, myocardia, or lung; with the exception of calcified lymph nodes and asymptomatic arterial or cartilage/tendon calcification.

3.29f Unable or unwilling to swallow Pemigatinib and keep a medication diary, or significant gastrointestinal disorder(s) that could interfere with absorption, metabolism or excretion of Pemigatinib per the discretion of the investigator.

3.29g Any of the following because this study involves an investigational agent whose genotoxic, mutagenic and teratogenic effects on the developing fetus and newborn are unknown:

- Pregnant women.
- Nursing women.
- Women of childbearing potential or men able to father children who have a female partner of childbearing potential, who are unwilling to employ acceptable contraception as outlined in Appendix I.

3.29h Known history of human immunodeficiency (HIV) infection or positivity on immunoassay confirmed per local standards

Note: HIV test is not required for screening, but patients with a known history of HIV infection will be excluded.

3.29i Evidence of active hepatitis B (HBV) or hepatitis C (HCV) infection

3.29j Other known active malignancy ≤ 5 years prior to registration.

EXCEPTIONS: Non-melanotic skin cancer or carcinoma in situ of the cervix, provided there is no known active disease and no additional therapy for the condition is ongoing or required during the trial period

NOTE: Anti-estrogen/androgen therapy or bisphosphonates allowed.

3.29k Co-morbid systemic illness, other severe concurrent disease, or psychiatric illness/social situation which, in the judgment of the investigator, would make the patient inappropriate for entry into this study, limit compliance with study requirements, or interfere significantly with the proper assessment of safety and

toxicity of the prescribed regimen.

4.0 Test Schedule

4.1 Schedule of Assessments

Tests and Procedures	Screening	Active Monitoring					
		Cycle 1			Cycles 2[+]	End of Treatment	Safety[12]
	Days -28 to -1	Day 1[14]	Day 8 ±3 days[10]	Day 15 ±3 days[10]	Day 1 ±3 days[10]	Last day of treatment +14 days[11]	EOT +30-35 days
COLOMATE Companion Trial Recommendation Form(optional)[1]	X						
Demography and medical history	X						
Hepatitis B and C screening	X						
Vital Signs, ECOG performance status[2]	X	X	X	X	X	X	X
Physical exam, weight, height	X	X	X	X	X	X	X
Review AEs	X	X	X	X	X	X	X
Review prohibited meds[3]	X	X	X	X	X	X	X
Review patient medication diary			X	X	X	X	
Eye examination[R]	X				X[4] (every 3 cycles starting with c3d1)	X	

Tests and Procedures	Screening	Active Monitoring					
		Cycle 1			Cycles 2[+]	End of Treatment	Safety[12]
	Days -28 to -1	Day 1[14]	Day 8 ±3 days[10]	Day 15 ±3 days[10]	Day 1 ±3 days[10]	Last day of treatment +14 days[11]	EOT +30-35 days
CT or MRI[5]	X				X[5] (Restaging at end of cycles 3, 6, 9 etc.)	X[5]	
12-lead ECG[6]	X	X		X	X	X	X
Hematology and Serum Chemistries	X	X	X	X	X	X	X
Tumor Blood Biomarkers (CEA)[18]	X[18]	X			X		
Coagulation panel	X				X[15] (day 1 of c4, 7, 10 etc.)	X	
Parathyroid hormone (PTH)[R]	X	X			X	X	
Lipid panel[R]		X				X	
Mandatory Patient Questionnaire Booklet (Appendix V)[19]		X			X	X	
Urinalysis	X				X[16] (day 1 of c4, 7, 10 etc.)		

Tests and Procedures	Screening	Active Monitoring					
		Cycle 1			Cycles 2[+]	End of Treatment	Safety[12]
	Days -28 to -1	Day 1[14]	Day 8 ±3 days[10]	Day 15 ±3 days[10]	Day 1 ±3 days[10]	Last day of treatment +14 days[11]	EOT +30-35 days
Pregnancy test[7]	X[7]	X			X	X	
Blood sample-cfDNA Guardant360 (optional) [13,R]						X	
Mandatory archival tissue [8,R]	X						
Mandatory blood sample plasma [9,R]		X			X[9] (c2d1; then on d1 following restaging scans (c4, 7, 10, etc.)	X	
Mandatory blood sample-Whole blood[R]		X[17]					

Notes:

[R] Research funded (see Section 19).

1. If the subject is participating through the COLOMATE platform: The COLOMATE Companion Trial Recommendation Form must indicate that the patient has met molecular eligibility to screen. The patient must be registered to the companion study ≤30 days from date of COLOMATE Companion Trial Recommendation Form completion. If the COLOMATE Companion Trial Recommendation Form recommends a different companion study, patient may still enroll after approval by the study PI or designee. Form must be uploaded to supporting documentation folder in RAVE.

2. Vital Signs: Including heart rate, blood pressure and temperature (oral or tympanic) and ideally performed with subject in a recumbent or semirecumbent position after 5 minutes of rest.

3. Prohibited Medications : List of prohibited medications in section 9.11 should be reviewed at study entry and during treatment.

4. Comprehensive Eye Examination: To be performed by a qualified ophthalmologist at screening, once every 3 cycles starting with Cycle 3, Day 1 (±14 days); at EOT, and as clinically indicated. The eye examination should include a visual acuity test, slit-lamp examination, OCT, and funduscopy with digital imaging. Additional assessments should be performed if clinically relevant retinal findings are observed on ophthalmologic exams and in participants with reported visual AEs or change in visual acuity, if the events or changes are suspected to be of retinal origin. Every effort should be made to ensure that all subsequent examinations are performed by the same ophthalmologist.

5. Computed Tomography (CT) / Magnetic Resonance Imaging (MRI): Baseline evaluation of disease status by CT or MRI completed ≤ 28 days prior to registration. Baseline and subsequent scans to include imaging of the primary site of disease plus any additional sites of known or suspected disease. Re-scanning to occur every 9 weeks (minus 1-6 days) after initiating protocol treatment on Cycle 1, Day 1. (The minus 1-6 day scan window is intended for flexibility in scheduling re-scans during Week 3 of every 3rd cycle – i.e. re-scanning intended between Days 15-21 of Cycles 3, 6, 9, etc.)
   Same-day scanning on Day 1 of Cycles 4, 7, 10, etc. is discouraged but allowed, provided scan results receive appropriate RECIST review prior to initiating/continuing Pemigatinib in the new cycle of study treatment (e.g. first re-scan is discouraged, but permitted on Cycle 4, Day 1, provided RECIST review occurs prior to dispensing Pemigatinib for Cycle 4).
   CT/MRI required at EOT only if it has been >28 days since the previous CT/MRI.

6. Electrocardiogram (ECG): Single trace 12-lead ECGs on site equipment ideally performed with subject in a recumbent or semirecumbent position after 5 minutes of rest. ECGs will be interpreted locally by the site and used for subject management. Management decisions (e.g. include or exclude a subject or withdraw a subject from the study) based on an ECG finding such as "Abnormal, Clinically Significant" is the responsibility of the investigator, in consultation with the sponsor-investigator, as appropriate. Clinically notable abnormalities considered clinically significant by the investigator are to be reported as AEs.

7. Pregnancy Test: For all women of childbearing potential (WOCBP) – as defined in Appendix I – serum pregnancy test required during screening; and serum or urine pregnancy test to be conducted on Day 1 of Cycle 1 (before first dose of Pemigatinib), on Day 1 of every cycle, at the EOT visit, and additionally as medically indicated. If a urine pregnancy test is positive, the results should be confirmed with a serum pregnancy test: If the serum pregnancy test is negative after a urine test was positive, the investigator will assess the potential benefit/risk to the subject and determine whether it is in the subject's best interest to resume study drug and continue participation in the study.

8. Archival Tissue: Receipt of archival tumor tissue is not required for study registration and initiation of therapy. However, it is mandatory to receive the required tissue within 90 days from randomization. See section 17.0.

9. Mandatory Blood Sample-Plasma: Mandatory collection on Day 1, cycle 1; day 1, cycle 2; and on day 1 of each cycle which is immediately following radiographic assessment for re-staging (day 1 of cycles 4,7,10, etc.) and at EOT (see Section 14). Kits required for collection. Patient must be registered prior to collection.

10. Administrative Scheduling Window: After Cycle 1, Day 1, allowance of ±3 days for subsequent study visits is permitted for scheduling considerations other than adverse event management (e.g. holidays, weekends, inclement weather).

11. End-of -Treatment (EOT): Reasonable effort should be made to complete End-of-Treatment (EOT) procedures on the day it is decided a patient will no longer receive Pemigatinib. These procedures must be completed subsequent to and not later than 14 days after investigator decision to permanently discontinue Pemigatinib and prior to any subsequent anti-cancer therapy. CT/MRI required at EOT only if it has been >28 days since the previous CT/MRI.

12. Safety Follow-Up: One-month follow-up (1M F/U) visit to be performed 30 to 35 days after the EOT visit. (In the event EOT visit not performed, then the safety follow-up visit is to be performed 30-35 days after the last dose of Pemigatinib.) Any adverse event in the Safety Follow-up will be recorded on the adverse event assessment form in the last cycle of treatment. The Safety Follow-up is recommended by the study protocol, but no data collection is required other than adverse event assessment.

13. Patients have the option to complete cfDNA testing at study completion and will be allowed to re-register with protocol ACCRU-GI-1611. Site will access results by creating an account at https://portal.guardanthealth.com. Sites will be notified by Guardant Health via email when results are available in the portal. See section 13.8. See detailed instructions on the ACCRU website under Manuals and Forms. Kits are required for this collection.

14. If screening tests occur ≤ 3 days prior to C1 D1, they do not have to be repeated prior to dosing.

15. Coagulation Panel: to be done at screening, restaging (day 1 of cycles 4, 7, 10 etc.) and end of treatment.

16. Urinalysis: to be done at screening and restaging (day 1 of cycles 4, 7, 10, etc.).

17. Mandatory blood sample-Whole blood: May occur prior to treatment on cycle 1 day 1. Refer to section 14.0

18. Tumor Blood Biomarkers (CEA): to be done ≤7 days prior to registration, and day 1 of every cycle thereafter.

19. Patient questionnaire will be completed at baseline, day 1 of each cycle, and end of treatment (within 30 days of last dose as noted in appendix V). Patient questionnaire booklets must be used; copies are not acceptable for this submission. The booklet order form is located on the ACCRU website under Manuals and Forms.

4.2 Local Laboratory Tests: Required Analytes

<table>
<tr><th>Serum Chemistries</th><th>Hematology</th><th>Urinalysis with Microscopic Examination</th><th>Hepatitis Screening</th><th>Coagulation</th></tr>
<tr><td rowspan="3">Albumin
Alkaline phosphatase
ALT
AST
Bicarbonate
Blood urea nitrogen
Calcium
Chloride
Creatinine
Glucose
Lactate dehydrogenase
Phosphate
Potassium
Sodium
Total bilirubin
Total protein
Uric acid
Vitamin D (25-hydroxyvitamin D and 1,25-dihidroxyvitamin D)</td><td rowspan="3">Complete blood count, including:
• Hemoglobin
• Hematocrit
• Platelet count
• Red blood cell count
• White Blood cell count

Differential count, including:
• Basophils
• Eosinophils
• Lymphocytes
• Monocytes
• Neutrophils

<u>Absolute values must be provided for</u>: WBC differential laboratory results:
Lymphocytes
Neutrophils</td><td>Color and appearance
pH and specific gravity
Bilirubin
Glucose
Ketones
Leukocytes
Nitrite
Occult blood
Protein
Urobilinogen</td><td>Hepatitis B surface antigen
Hepatitis B surface antigen antibody
Hepatitis B core antibody HCV antibody

NOTE: If any of the above are positive, then HBV-DNA, HCV-RNA to assess risk of reactivation.</td><td>PT
PTT
INR</td></tr>
<tr><th>Lipid Panel</th><th>Other</th><th>Pregnancy Testing</th></tr>
<tr><td>Total cholesterol
Triglycerides
LDL
HDL</td><td>Endocrine: Parathyroid hormone</td><td>Required only for female subjects of childbearing potential. Serum pregnancy test at screening; and serum or urine pregnancy test on Day 1 of Cycle 1 (before first dose of Pemigatinib), on Day 1 of every cycle, and at EOT.</td></tr>
</table>

5.0 Grouping Factors

Type of FGFR alteration:

- Mutations Vs.
- Translocations/Fusion Vs.
- Amplifications

6.0 Registration Procedures

6.1 Registration Procedures

6.11 To register a patient, fax (507-284-0885) a completed eligibility checklist to the Academic and Community Cancer Research United (ACCRU) Registration Office between 8 a.m. and 4:30 p.m. central time Monday through Friday.

Only required if patient is participating in ACCRU-GI-1611.NOTE: ACCRU-GI-1611 (COLOMATE) treating physician will provide COLOMATE Companion Trial Recommendation Form which will include the Patient ID that must be used for this study.

6.12 Mandatory

A mandatory correlative research component is part of this study. The patient will be automatically registered onto this component (see Sections 3.0, 14.0 and 17.0).

Optional

An optional correlative research component is part of this study. There will be an option to select if the patient is to be registered onto this component (see Section 14.0).

- Patient has/has not given permission to give his/her blood sample for research testing.

6.13 Documentation of IRB approval must be on file in the Registration Office before an investigator may register any patients. Approvals should be uploaded using the online ACCRU Regulatory Management System (ARMS).

In addition to submitting initial IRB approval documents, ongoing IRB approval documentation must be on file (no less than annually) with ACCRU. Approvals should be uploaded using the online ACCRU Regulatory Management System (ARMS). If the necessary documentation is not submitted in advance of attempting patient registration, the randomization will not be accepted and the patient may not be enrolled in the protocol until the situation is resolved.

Submission of annual IRB approvals is required until the study has been closed through your IRB.

6.14 Prior to accepting the registration/randomization, the registration/randomization application will verify the following:
- IRB approval at the registering institution
- Patient eligibility
- Existence of a signed consent form
- Existence of a signed authorization for use and disclosure of protected health information

6.15 At the time of registration, the following will be recorded:

- Patient has/has not given permission to store and use his/her blood sample(s) for future research to learn about, prevent, or treat cancer.
- Patient has/has not given permission to store and use his/her blood sample(s) for future research to learn, prevent, or treat other health problems (for example: diabetes, Alzheimer's disease, or heart disease).
- Patient has/has not given permission to store and use his/her tissue sample(s) for future research to learn about, prevent, or treat cancer.
- Patient has/has not given permission to store and use his/her tissue sample(s) for future research to learn, prevent, or treat other health problems (for example: diabetes, Alzheimer's disease, or heart disease).
- Patient has/has not given permission for ACCRU to give his/her sample(s) to outside researchers.

6.16 Treatment cannot begin prior to registration and must begin ≤10 days after registration.

6.17 Pretreatment tests/procedures (see Section 4.0) must be completed within the guidelines specified on the test schedule.

6.18 All required baseline symptoms (see Section 10.5) must be documented and graded.

6.19a Treatment on this protocol must commence at an ACCRU institution under the supervision of a medical oncologist.

6.19b Blood draw kit is available on site.

Note: Guardant 360 kit does not need to be on site at the time of registration.

6.19c Patient questionnaire booklet is available on site; copies are not acceptable for this submission.

7.0 Protocol Treatment

7.1 Treatment Schedule

Agent	Dose	Route	Schedule
Pemigatinib	13.5 mg<br>(dose level 0)	Oral	Once daily (QD),<br>on Days 1-21 of cycle 1
Pemigatinib	18 mg*	Oral	Once daily (QD),<br>on Days 1-21 of cycles 2-35*

During each 21-day dosing cycle, Pemigatinib is scheduled to be administered on days 1-21 of a 21 day cycle

Each dose of Pemigatinib should be taken by mouth with a glass of water immediately upon rising or after a 2-hour fast. After dosing, the patient should refrain from eating for 1 hour.

If the subject vomits after taking study drug, the subject should not take another dose that day. If a dose of Pemigatinib is missed by more than 4 hours, that dose should be skipped and the next scheduled dose should be administered at the usual time.

***All of the following criteria must be met in order to increase dose level beginning at cycle 2**

- Participant does not reach the target serum phosphate level of > 5.5 mg/dL at any time during Cycle 1.
- Patient is compliant with taking study drug.
- Patient does not experience an ongoing Grade 2 or higher treatment-related AEs during cycle 1.
- If dose level escalated, please remember to report as modified treatment in Rave.

7.2 If all criteria for dose increase are not met patient will remain at dose level 0. Dose may be increased at a future cycle if patient meets criteria. Note: If dose escalated, please remember to report as modified treatment in Rave.

7.3 Patients can be instructed in Pemigatinib administration techniques and granted treatment independence with nursing staff approval.

7.4 For this protocol, the patient must return to the consenting ACCRU institution for evaluation during treatment and during observation (Active Monitoring Phase) according to the table in Section 4.0.

7.5 Patients will be required to keep a medication diary for Pemigatinib using the diary in appendix III. Sites will be required to upload medication diary to RAVE at the end of each cycle.

8.0 Dosage Modification Based on Adverse Events

For all adverse events reported in this study considered by the investigator as possibly, probably

or definitely related to Pemigatinib, the following dose modification guidance should be applied.

Adverse events considered by the investigator as definitely related to something other than Pemigatinib, or transient (≤ 72 hours) abnormal laboratory values without associated signs or symptoms deemed clinically significant by the investigator, may be exempt from dose-reduction rules unless otherwise requested or required by the sponsor-investigator.

ALERT: ADR reporting may be required for some adverse events (See Section 10)

8.1 Dose Level Reductions (if needed for management of potential adverse event)

Dose Level	Pemigatinib
1	18 mg PO QD
0	13.5 mg PO QD
-1	9 mg PO QD

* All patients are scheduled to initiate Pemigatinib at Dose Level 0.
See section 7.1 for dose escalation information.
Note: If dose escalated, please report as modified treatment in Rave.

8.2 Dose Modifications

Use the NCI Common Terminology Criteria for Adverse Events (CTCAE) Version 5.0 dated November 27, 2017* unless otherwise specified		
**ADVERSE EVENT**	**AGENT**	**ACTION**
**Chemistry**		
AST and/or ALT > 5.0 x ULN and/or ALP > 5.0 x ULN<br><br>Note: In patients with liver or bone metastasis-related elevations at baseline, contact sponsor-investigator to discuss clinical management and possible dose reductions.	Pemigatinib	**Step 1:** *Hold/Delay Pemigatinib* up to 2 weeks (14 days) until the toxicity has resolved to ≤ Grade 1 except by written approval from the sponsor-investigator.<br><br>**Step 2:** If assessed by the investigator as not related or unlikely related to Pemigatinib, then restart Pemigatinib at *same dose*; monitor as clinically indicated.<br><br>**Step 2:** If assessed by the investigator as possibly, probably or definitely related to Pemigatinib, then restart Pemigatinib at the *next lower dose level*; monitor as clinically indicated.

ANC is 0.5 x 10^9/L to < 1.0 × 10^9/L (grade 3)<br><br>Platelet count is 50 × 10^9/L to < 75 × 10^9/L (grade 2)	Pemigatinib	**Step 1:** Interrupt study drug up to 2 weeks (14 days) until the toxicity has resolved to Grade 1 or pretherapy baseline.<br>**Step 2:** Restart study drug at same dose and monitor as clinically indicated.<br><br>*If neutropenia is rapidly reversible after stopping study drug, subjects will be allowed to stay on their dose as long as ANC > 1.0 × 10^9/L.
Grade 4 ANC (< 0.5 × 10^9/L)<br><br>Grade 3 ANC with an oral temperature of at least 38.5°C OR with Grade 3 infection<br><br>Platelet count is < 50 × 10^9/L (grade 3 or 4)	Pemigatinib	**Step 1:** Interrupt study drug up to 2 weeks (14 days) until resolved to Grade 1.<br>**Step 2:** If assessed as related to study drug, restart study drug at next lower dose level, except if Grade 4 neutropenia resolves in ≤ 7 days, in which case restart at the same dose.
**Other Toxicities**		
Any Grade 1 or other Grade 2 toxicity	Pemigatinib	Continue Pemigatinib; treat the toxicity and monitor as clinically indicated.
Any other Grade 3 toxicity, if clinically significant and not manageable by supportive care.	Pemigatinib	**Step 1:** <u>*Hold/Delay*</u> Pemigatinib up to 2 weeks (14 days), until toxicity resolves to ≤ Grade 1.<br><br>**Step 2:** If assessed by the investigator as not related or unlikely related to Pemigatinib, then restart Pemigatinib at <u>*same dose*</u>; monitor as clinically indicated.<br><br>**Step 2:** If assessed by the investigator as possibly, probably or definitely related to Pemigatinib, then restart Pemigatinib at the <u>*next lower dose level*</u>; monitor as clinically indicated.
Any recurrent Grade 3 toxicity after 2 dose reductions.	Pemigatinib	<u>*Permanently discontinue*</u> Pemigatinib and initiate post-treatment follow-up per Protocol. (Exceptions require written approval of sponsor-investigator.)

Any other Grade 4 toxicity, if clinically significant	Pemigatinib	*Permanently discontinue* Pemigatinib and initiate post-treatment follow-up per Protocol.

* Located at http://ctep.cancer.gov/protocolDevelopment/electronic_applications/ctc.htm

** The following describe actions in the Action column:

- Hold/Delay = Treatment can be made up (see Section 8.3) as part of this cycle;
- Discontinue = Treatment is totally stopped.

Hyperphosphatemia is an expected on-target pharmacologic effect of FGFR inhibition. Hyperphosphatemia should be managed with diet modifications, phosphate binders and diuretics, or a dose reduction per the recommendations in the below table:

**Recommended Approach for Hyperphosphatemia Management**			
**Serum Phosphate Level**	**Supportive Care**	**Guidance for Interruption/Discontinuation of Pemigatinib**	**Guidance for Restarting Pemigatinib**
> 5.5 mg/dL and ≤ 7 mg/dL	Initiate a low-phosphate diet.	No action.	Not applicable.
> 7 mg/dL and ≤ 10 mg/dL	Initiate/continue a low-phosphate diet and initiate phosphate-binding therapy once serum phosphate level is > 7 mg/dL.<br><br>Monitor serum phosphate at least twice a week and adjust the dose of binders as needed; continue to monitor serum phosphate at least twice a week until return to normal range.	If serum phosphate level continues to be > 7 mg/dL and ≤ 10 mg/dL with concomitant phosphate-binding therapy for 2 weeks, or if there is recurrence of serum phosphate level in this range, *hold/delay* Pemigatinib for up to 2 weeks	Restart Pemigatinib at the *same dose* when serum phosphate is < 7 mg/dL.<br><br>If serum phosphate level recurs at > 7 mg/dL, restart Pemigatinib *with dose reduction*.

> 10 mg/dL	Continue to maintain a low-phosphate diet, adjust phosphate-binding therapy, and start/continue phosphaturic agent.<br><br>Continue to monitor serum phosphate at least twice a week until return to normal range.	If serum phosphate level is > 10 mg/dL for 1 week following phosphate-binding therapy and low phosphate diet, *hold/delay Pemigatinib*.<br><br>If there is recurrence of serum phosphate level in this range following 2 dose reductions, *permanently discontinue* Pemigatinib.	Restart Pemigatinib *at reduced dose* with phosphate binders when serum phosphate is < 7 mg/dL.

** The following describe actions above:

- Hold/Delay = Treatment can be made up (see Section 8.3) as part of this cycle;
- Discontinue = Treatment is totally stopped.

8.3 For reasons that are consistent with the Dose Modifications outlined above in Section 8.2, Pemigatinib treatment that is held/delayed for purpose of adverse event management can be "made up" within a given cycle:

- The scheduled duration of a cycle is 21 days (3 weeks).
- For purpose of accommodating an established Pemigatinib dose hold/delay, the number of days in any given cycle may be increased up to 14 total days, in order to accommodate a maximum cycle length of 35 days (5 weeks).

  Note: The purpose of this guidance is not to accommodate re-dosing in the absence of an established Pemigatinib dose hold/delay. For example, inadvertently missed or vomited doses of Pemigatinib are not to be "made up" within the cycle, but rather should be omitted from the cycle (and noted as such in the patient-completed medication diary).
- Within every given cycle, an Pemigatinib dose hold/delay is permitted, with a maximum duration of no more than 14 total days in each cycle (regardless of whether such 14 total days within any given cycle are consecutive or cumulative days).
- In all cases, a patient may receive no more than 21 total doses of Pemigatinib in a single cycle.
- Dose re-escalation only allowed after discussion with study PI and sponsor, if clinically indicated.
- If dosing is delayed greater than 42 days, patient should be removed from treatment, and proceed to event monitoring.

9.0 Ancillary Treatment/Supportive Care

9.1 Prohibited Medications

9.11 The following medications and measures are PROHIBITED:

- Concomitant administration of potent CYP3A4 inhibitors and inducers and moderate CYP3A4 inducers. Based on the low overall bioavailability of topical ketoconazole, there are no restrictions on topical ketoconazole. See appendix II for a list of potent CYP3A4 inhibitors and inducers.
- Any concomitant use of a selective FGFR inhibitor (other than Pemigatinib).
- Investigational study drug for any indication.
- Use of any anticancer medications (other than Pemigatinib).

Restricted and Cautionary Medications

9.12 The use of mild or moderate CYP3A4 inhibitors or inducers should involve careful monitoring.

9.13 The pH level of stomach acid impacts the absorption of Pemigatinib. As a result, *limited use* of proton pump inhibitors or antacids while on study is recommended.

9.14 Calcium-based phosphate binding medications while on study should be avoided or, if medically necessary and unavoidable, used with caution due to a concern for soft tissue mineralization.

9.2 Supportive Care

9.21 Hyperphosphatemia is an expected on-target pharmacologic effect of FGFR inhibition. Hyperphosphatemia should be managed with diet modifications, phosphate binders and diuretics, or a dose reduction per the recommendations in Section 8.

9.22 Patients should receive full supportive care while on this study. This includes blood product support, antibiotic treatment, and treatment of other newly diagnosed or concurrent medical conditions.

10.0 Adverse Event (AE) Reporting and Monitoring

The site principal investigator is responsible for reporting any/all adverse events to the sponsor as described within the protocol. Refer to the adverse event and serious adverse event sections of the protocol for detailed information.

The sponsor/sponsor-investigator is responsible for notifying FDA and all participating investigators in a written safety report of any of the following:

- Any suspected adverse reaction that is both serious and unexpected.
- Any findings from laboratory animal or *in vitro* testing that suggest a significant risk for human subjects, including reports of mutagenicity, teratogenicity, or carcinogenicity.
- Any findings from epidemiological studies, pooled analysis of multiple studies, or clinical studies, whether or not conducted under an IND and whether or not conducted by the sponsor, that suggest a significant risk in humans exposed to the drug.
- Any clinically important increase in the rate of a serious suspected adverse reaction over the rate stated in the protocol or Investigator's Brochure (IB).

Definitions

Adverse Event
Any untoward medical occurrence associated with the use of a drug in humans, whether or not considered drug related.

Suspected Adverse Reaction
Any adverse event for which there is a reasonable possibility that the drug caused the adverse event.

Expedited Reporting
Events reported to sponsor within 24 hours, 5 days or 10 days of study team becoming aware of the event.

Routine Reporting
Events reported to sponsor via case report forms

Events of Interest
Events that would not typically be considered to meet the criteria for expedited reporting, but that for a specific protocol are being reported via expedited means in order to facilitate the review of safety data (may be requested by the FDA or the sponsor).

10.1 Adverse Event Characteristics

CTCAE term (AE description) and grade: The descriptions and grading scales found in the revised NCI Common Terminology Criteria for Adverse Events (CTCAE) version 5.0 will be utilized for AE reporting. All appropriate treatment areas should have access to a copy of the CTCAE version 5.0. A copy of the CTCAE version 5.0 can be downloaded from the CTEP web site: (http://ctep.cancer.gov/protocolDevelopment/electronic_applications/ctc.htm)

a. Adverse event monitoring and reporting is a routine part of every clinical trial.

b. Identify the grade and severity of the event using the CTCAE version 5.0.

c. Determine whether the event is expected or unexpected (see Section 10.2).

d. Determine if the adverse event is related to the study intervention (agent, treatment or procedure) (see Section 10.3).

e. Determine whether the event must be reported as an expedited report. If yes, determine the timeframe/mechanism (see Section 10.4).

f. Determine if other reporting is required (see Section 10.5).

g. Note: All AEs reported via expedited mechanisms must also be reported via the routine data reporting mechanisms defined by the protocol (see Sections 10.5 and 18.0).

Each CTCAE term in the current version is a unique representation of a specific event used for medical documentation and scientific analysis and is a single MedDRA Lowest Level Term (LLT).
NOTE: A severe AE, as defined by the above grading scale, is NOT the same as serious AE which is defined in the table in Section 10.4.

10.2 Expected vs. Unexpected Events

Expected events - are those described within the Section 15.0 of the protocol, the study specific consent form, package insert (if applicable), and/or the investigator brochure, (if an investigator brochure is not required, otherwise described in the general investigational plan).

Unexpected adverse events or suspected adverse reactions are those not listed in Section 15.0 of the protocol, the study specific consent form, package insert (if applicable), or in the investigator brochure (or are not listed at the specificity or severity that has been observed); if an investigator brochure is not required or available, is not consistent with the risk information described in the general investigational plan.

Unexpected also refers to adverse events or suspected adverse reactions that are mentioned in the investigator brochure as occurring with a class of drugs but have not been observed with the drug under investigation.

10.3 Assessment of Attribution

When assessing whether an adverse event is related to a medical treatment or procedure, the following attribution categories are utilized:

Definite - The adverse event *is clearly related* to the agent(s).
Probable - The adverse event *is likely related* to the agent(s).
Possible - The adverse event *may be related* to the agent(s).
Unlikely - The adverse event *is doubtfully related* to the agent(s).
Unrelated - The adverse event *is clearly NOT related* to the agent(s).

Events determined to be possibly, probably or definitely attributed to a

medical treatment suggest there is evidence to indicate a causal relationship between the drug/device and the adverse event.

The following hospitalizations are not considered to be SAEs because there is no "adverse event" (i.e., there is no untoward medical occurrence) associated with the hospitalization:

- Hospitalizations for respite care
- Planned hospitalizations required by the protocol or to administer protocol directed treatment
- Hospitalization planned before informed consent (where the condition requiring the hospitalization has not changed post study drug administration)
- Hospitalization for administration of study drug
- Hospitalization for routine maintenance of a device (e.g., battery replacement) that was in place before study entry

*Report any clinically important increase in the **rate** of a serious suspected adverse reaction (at your study) site over that which is listed in the protocol or investigator brochure as an expedited event.

*Report an expected event that is greater in severity or specificity than expected as an expedited event

*An investigational agent/intervention might exacerbate the expected AEs associated with a commercial agent. Therefore, if an expected AE (for the commercial agent) occurs with a higher degree of severity or specificity, expedited reporting is required.

A list of known/expected AEs is reported in the investigator brochure, package insert or the literature, including AEs resulting from a drug overdose.

10.331 Death

- Any death occurring within 30 days of the last dose, regardless of attribution to an agent/intervention under an IND/IDE requires expedited reporting within 24-hours.
- Any death occurring greater than 30 days with an attribution of possible, probable, or definite to an agent/intervention under an IND/IDE requires expedited reporting within 24-hours.
- Reportable categories of Death
    - Death attributable to a CTCAE term.

- Death Neonatal: A disorder characterized by cessation of life during the first 28 days of life.

- Death NOS: A cessation of life that cannot be attributed to a CTCAE term associated with Grade 5.

- Sudden death NOS: A sudden (defined as instant or within one hour of the onset of symptoms) or an unobserved cessation of life that cannot be attributed to a CTCAE term associated with Grade 5.

- Death due to progressive disease should be reported as **Grade 5 "Neoplasms benign, malignant and unspecified (including cysts and polyps) – Other (Progressive Disease)"** under the system organ class (SOC) of the same name. Evidence that the death was a manifestation of underlying disease (e.g., radiological changes suggesting tumor growth or progression: clinical deterioration associated with a disease process) should be submitted.

10.332 Secondary Malignancy

- A *secondary malignancy* is a cancer caused by treatment for a previous malignancy (e.g., treatment with investigational agent/intervention, radiation or chemotherapy). A secondary malignancy is not considered a metastasis of the initial neoplasm.

- All secondary malignancies that occur following treatment with an agent under an IND/IDE to be reported. Three options are available to describe the event:

    - Leukemia secondary to oncology chemotherapy (e.g., Acute Myeloctyic Leukemia [AML])

    - Myelodysplastic syndrome (MDS)

    - Treatment-related secondary malignancy

- Any malignancy possibly related to cancer treatment (including AML/MDS) should also be reported via the routine reporting mechanisms outlined in each protocol.

10.333 Second Malignancy

- A second malignancy is one unrelated to the treatment of a prior malignancy (and is NOT a metastasis from the initial malignancy). Second malignancies require ONLY routine reporting.

10.334 Pregnancy

An “Initial Pregnancy Report” must be completed in full and faxed to the ACCRU SAE coordinator at (507) 284-9628 within 24 hours of discovery of a pregnancy of a subject who has taken the Incyte product (INCB054828) or the pregnancy of a partner for a subject who has taken the Incyte product. The “Follow-up Pregnancy Report Form” must be completed and faxed to the ACCRU SAE coordinator at (507) 284-9628 within 30 days after delivery, so that Incyte is provided with information regarding the outcome of the pregnancy. The ACCRU SAE coordinator will forward all pregnancy reports to Incyte. If the pregnancy results in any events which meet the serious criteria (i.e., miscarriage or termination), the SAE reporting process needs to be followed and the timelines associated with an SAE should be followed.

“Initial Pregnancy Report” and “Follow-up Pregnancy Report Form” can be found on the ACCRU website.

Prior to obtaining private information about a pregnant woman and her infant, the investigator must obtain consent from the pregnant woman and the newborn infant’s parent or legal guardian before any data collection can occur. A consent form will need to be submitted to the IRB for these subjects if a pregnancy occurs. If informed consent is not obtained, no information may be collected.

In cases of fetal death, miscarriage or abortion the mother is the patient. In cases where the child/fetus experiences a serious adverse event other than fetal death, the child/fetus is the patient.

NOTE: When submitting ACCRU Adverse Event Report reports for “Pregnancy”, “Pregnancy loss”, or “Neonatal loss”, the potential risk of exposure of the fetus to the investigational agent(s) or chemotherapy agent(s) should be documented in the “Description of Event” section. Include any available medical documentation.

10.4 Expedited Adverse Event Reporting Requirements for IND/IDE Agents

10.41 Early Phase 2 Studies: Expedited Reporting via the **ACCRU Adverse Event Expedited Report Form** for Adverse Events That Occur Within 30 Days[1] of the Last Dose of the Investigational Agent

FDA REPORTING REQUIREMENTS FOR SERIOUS ADVERSE EVENTS (21 CFR Part 312)

NOTE: Investigators **MUST** immediately report to the sponsor **ANY** Serious Adverse Events, whether or not they are considered related to the investigational agent(s)/intervention (21 CFR 312.64)

An adverse event is considered serious if it results in **ANY** of the following outcomes:

1) Death
2) A life-threatening adverse event
3) An adverse event that results in inpatient hospitalization or prolongation of existing hospitalization for ≥ 24 hours
4) A persistent or significant incapacity or substantial disruption of the ability to conduct normal life functions
5) A congenital anomaly/birth defect.
6) Important Medical Events (IME) that may not result in death, be life threatening, or require hospitalization may be considered serious when, based upon medical judgment, they may jeopardize the patient or subject and may require medical or surgical intervention to prevent one of the outcomes listed in this definition. (FDA, 21 CFR 312.32; ICH E2A and ICH E6).

ALL SERIOUS adverse events that meet the above criteria MUST be immediately reported to the sponsor within the timeframes detailed in the table below.

Hospitalization	Grade 1 and Grade 2 Timeframes	Grade 3-5 Timeframes
Resulting in Hospitalization ≥ 24 hrs	7 Calendar Days	24-Hour; 3 Calendar Days
Not resulting in Hospitalization ≥ 24 hrs	Not required	

Expedited AE reporting timelines are defined as:

- "24-Hour; 3 Calendar Days" - The AE must initially be reported within 24 hours of learning of the AE, followed by a complete expedited report within 3 calendar days of the initial 24-hour report.
- "7 Calendar Days" - A complete expedited report on the AE must be submitted within 7 calendar days of learning of the AE.

[1]Serious adverse events that occur more than 30 days after the last administration of investigational agent/intervention and have an attribution of possible, probable, or definite require reporting as follows:

Expedited 24-hour notification followed by complete report within 3 calendar days for:

- All Grade 3, 4, and Grade 5 AEs

Expedited 7 calendar day reports for:

- Grade 2 AEs resulting in hospitalization or prolongation of hospitalization

[2] For studies using PET or SPECT IND agents, the AE reporting period is limited to 10 radioactive half-lives, rounded UP to the nearest whole day, after the agent/intervention was last administered. Footnote "1" above applies after this reporting period.

Effective Date: May 5, 2011

Special Instructions:

- Follow site-specific reporting guidelines.
    - Submit the ACCRU Adverse Event Expedited Report Form to ACCRU Safety via email at: ACCRUSAFETY@mayo.edu. ACCRU Safety will forward to Incyte via email to safetyreporting@incyte.com
- SAE reports should be for a single subject.
- ACCRU Safety will forward to ACCRU IND Coordinator (INDINFORMATION@mayo.edu) as appropriate. The ACCRU IND Coordinator will assist the sponsor-investigator in notifying the FDA if required.

10.5 Other Required Reporting

10.51 Unanticipated Problems Involving Risks to Subjects or Others (UPIRTSOS) in general, include any incident, experience, or outcome that meets **all** of the following criteria:

1. Unexpected (in terms of nature, severity, or frequency) given (a) the research procedures that are described in the protocol-related documents, such as the IRB-approved research protocol and informed consent document; and (b) the characteristics of the subject population being studied;

2. Related or possibly related to participation in the research (in this guidance document, possibly related means there is a reasonable possibility that the incident, experience, or outcome may have been caused by the procedures involved in the research); and

3. Suggests that the research places subjects or others at a greater risk of harm (including physical, psychological, economic, or social harm) than was previously known or recognized.

Some unanticipated problems involve social or economic harm instead of the physical or psychological harm associated with adverse events. In other cases, unanticipated problems place subjects or others at increased *risk* of harm, but no harm occurs.

Note: If there is no language in the protocol indicating that pregnancy is not considered an adverse experience for this trial, and if the consent form does not indicate that subjects should not get pregnant/impregnate others, then any pregnancy in a subject/patient or a male patient's partner (spontaneously reported) which occurs during the study or within 120 days of completing the study should be reported as a UPIRTSO.

If the event meets the criteria for an UPIRTSO, submit to your IRB as required by your institutional policies.

10.52 Baseline and Adverse Events Evaluations

The following pre-treatment symptoms/conditions are to be graded at baseline and adverse events are to be graded at each evaluation using CTCAE v5.0 grading.

System Organ Class (SOC)	Adverse event/Symptoms	Baseline	Each evaluation
General disorders and administration site conditions	Fatigue	X	X
Gastrointestinal disorders	Nausea	X	X
Gastrointestinal disorders	Vomiting	X	X
Gastrointestinal disorders	Diarrhea	# of stools	X
Metabolism and nutrition disorders	Anorexia	X	X
Metabolism and nutrition disorders	Hyperphosphatemia	X	X
Investigations	Alanine aminotransferase increased	X	X
Investigations	Aspartate aminotransferase increased	X	X
Investigations	Alkaline phosphatase increased	X	X
Investigations	Blood bilirubin increased	X	X
Investigations	Platelet count decreased	X	X
Investigations	Neutrophil count decreased	X	X
Blood and lymphatic system disorders	Anemia	X	X

10.53 Case Report Forms - Academic and Community Cancer Research United (ACCRU)

Submit the following AEs not specified in Section 10.5

10.531 Grade 2 AEs deemed *possibly, probably, or definitely* related to the study treatment or procedure.

10.532 Grade 3 and 4 AEs regardless of attribution to the study treatment or procedure.

10.533 Grade 5 AEs (Deaths)

10.5331 Any death within 30 days of the patient's last study treatment or procedure regardless of attribution to the study treatment or procedure.

10.5332 Any death more than 30 days after the patient's last study treatment or procedure that is felt to be at least possibly treatment related must also be submitted as a Grade 5 AE, with a CTCAE type and attribution assigned.

11.0 Treatment Evaluation Using RECIST Guideline

NOTE: This study uses protocol RECIST v1.1 template dated 2/16/2011. See the footnote for the table regarding measureable disease in Section 11.44, as it pertains to data collection and analysis.

Response and progression will be evaluated in this study using the new international criteria proposed by the revised Response Evaluation Criteria in Solid Tumors (RECIST) guidelines (version 1.1) (Eisenhauer et al., 2009). Changes in the largest diameter (unidimensional measurement) of the tumor lesions and the short axis measurements in the case of lymph nodes are used in the RECIST guideline.

11.1 Schedule of Evaluations: For the purposes of this study, patients should be reevaluated every 9 weeks.

11.2 Definitions of Measurable and Non-Measurable Disease

11.21 Measurable Disease

11.211 A non-nodal lesion is considered measurable if its longest diameter can be accurately measured as ≥2.0 cm with chest x-ray, or as ≥1.0 cm with CT scan, or MRI.

11.212 A superficial non-nodal lesion is measurable if its longest diameter is ≥ 1.0 cm in diameter as assessed using calipers (e.g. skin nodules) or imaging. In the case of skin lesions, documentation by color photography, including a ruler to estimate the size of the lesion, is recommended.

11.213 A malignant lymph node is considered measurable if its short axis is $\geq$1.5 cm when assessed by CT scan (CT scan slice thickness recommended to be no greater than 5 mm).

NOTE: Tumor lesions in a previously irradiated area are not considered measurable disease.

11.22 Non-Measurable Disease

11.221 All other lesions (or sites of disease) are considered non-measurable disease, including pathological nodes (those with a short axis ≥1.0 to <1.5 cm). Bone lesions, leptomeningeal disease, ascites, pleural/pericardial effusions, lymphangitis cutis/pulmonis, inflammatory breast disease, and abdominal masses (not followed by CT or MRI), are considered as non-measurable as well.

Note: ‘Cystic lesions’ thought to represent cystic metastases can be considered as measurable lesions, if they meet the definition of measurability described above. However, if non-cystic lesions are present in the same patient, these are preferred for selection as target lesions. In addition, lymph nodes that have a short axis <1.0 cm are considered non-pathological (i.e., normal) and should not be recorded or followed.

11.3 Guidelines for Evaluation of Measurable Disease

11.31 Measurement Methods:

- All measurements should be recorded in metric notation (i.e., decimal fractions of centimeters) using a ruler or calipers.

- The same method of assessment and the same technique must be used to characterize each identified and reported lesion at baseline and during follow-up. For patients having only lesions measuring at least 1 cm to less than 2 cm must use CT imaging for both pre- and post-treatment tumor assessments.

- Imaging-based evaluation is preferred to evaluation by clinical examination when both methods have been used at the same evaluation to assess the antitumor effect of a treatment.

11.32 Acceptable Modalities for Measurable Disease:

- Conventional CT and MRI: This guideline has defined measurability of lesions on CT scan based on the assumption that CT slice thickness is 5 mm or less. If CT scans have slice thickness greater than 5 mm, the minimum size for a measurable lesion should be twice the slice thickness.

- As with CT, if an MRI is performed, the technical specifications of the scanning sequences used should be optimized for the evaluation of the type and site of disease. The lesions should be measured on the same pulse sequence. Ideally, the same type of scanner should be used and the image

acquisition protocol should be followed as closely as possible to prior scans. Body scans should be performed with breath-hold scanning techniques, if possible.

11.33 Measurement at Follow-up Evaluation:

- The cytological confirmation of the neoplastic origin of any effusion that appears or worsens during treatment when the measurable tumor has met criteria for response or stable disease is mandatory to differentiate between response or stable disease (an effusion may be a side effect of the treatment) and progressive disease.

- Cytologic and histologic techniques can be used to differentiate between PR and CR in rare cases

11.4 Measurement of Effect

11.41 Target Lesions & Target Lymph Nodes

- Measurable lesions (as defined in Section 11.21) up to a maximum of 5 lesions, representative of all involved organs, should be identified as "Target Lesions" and recorded and measured at baseline. <u>These lesions can be non-nodal or nodal (as defined in 11.21)</u>, where no more than 2 lesions are from the same organ and no more than 2 malignant nodal lesions are selected.

  Note: If fewer than 5 target lesions and target lymph nodes are identified (as there often will be), there is no reason to perform additional studies beyond those specified in the protocol to discover new lesions.

- Target lesions and target lymph nodes should be selected on the basis of their size, be representative of all involved sites of disease, but in addition should be those that lend themselves to reproducible repeated measurements. It may be the case that, on occasion, the largest lesion (or malignant lymph node) does not lend itself to reproducible measurements in which circumstance the next largest lesion (or malignant lymph node) which can be measured reproducibly should be selected.

- Baseline Sum of Dimensions (BSD): A sum of the longest diameter for all target lesions plus the sum of the short axis of all the target lymph nodes will be calculated and reported as the baseline sum of dimensions (BSD). The BSD will be used as reference to further characterize any objective tumor response in the measurable dimension of the disease.

- Post-Baseline Sum of the Dimensions (PBSD): A sum of the longest diameter for all target lesions plus the sum of the short axis of all the target lymph nodes will be calculated and reported as the post-baseline sum of dimensions (PBSD). If the radiologist is able to provide an actual measure for the target lesion (or target lymph node), that should be recorded, even if it is below 0.5 cm. If the target lesion (or target lymph node) is believed to be present and is faintly seen but too small to measure, a default value of 0.5 cm should be

assigned. If it is the opinion of the radiologist that the target lesion or target lymph node has likely disappeared, the measurement should be recorded as 0 cm.

- The minimum sum of the dimensions (MSD) is the minimum of the BSD and the PBSD.

11.42 Non-Target Lesions & Non-Target Lymph Nodes

Non-measurable sites of disease (Section 11.22) are classified as non- target lesions or non-target lymph nodes and should also be recorded at baseline. These lesions and lymph nodes should be followed in accord with 11.433.

11.43 Response Criteria

11.431 All target lesions and target lymph nodes followed by CT/MRI must be measured on re-evaluation at evaluation times specified in Section 11.1. Specifically, a change in objective status to either a PR or CR cannot be done without re-measuring target lesions and target lymph nodes.

Note: Non-target lesions and non-target lymph nodes should be evaluated at each assessment, especially in the case of first response or confirmation of response. In selected circumstances, certain non-target organs may be evaluated less frequently. For example, bone scans may need to be repeated only when complete response is identified in target disease or when progression in bone is suspected.

11.432 Evaluation of Target Lesions

- Complete Response (CR): All of the following must be true:
  - Disappearance of all target lesions.
  - Each target lymph node must have reduction in short axis to <1.0 cm.
- Partial Response (PR): At least a 30% decrease in PBSD (sum of the longest diameter for all target lesions plus the sum of the short axis of all the target lymph nodes at current evaluation) taking as reference the BSD (*see* Section 11.41).
- Progression (PD): At least one of the following must be true:

- At least one new malignant lesion, which also includes any lymph node that was normal at baseline (< 1.0 cm short axis) and increased to ≥ 1.0 cm short axis during follow-up.

a. At least a 20% increase in PBSD (sum of the longest diameter for all target lesions plus the sum of the short axis of all the target lymph nodes at current evaluation) taking as reference the MSD (Section 11.41). In addition, the PBSD must also demonstrate an absolute increase of at least 0.5 cm from the MSD.

- Stable Disease (SD): Neither sufficient shrinkage to qualify for PR, nor sufficient increase to qualify for PD taking as reference the MSD.

11.433 Evaluation of Non-Target Lesions & Non-target Lymph Nodes

- Complete Response (CR): All of the following must be true:

  a. Disappearance of all non-target lesions.

  b. Each non-target lymph node must have a reduction in short axis to <1.0 cm.

- Non-CR/Non-PD: Persistence of one or more non-target lesions or non-target lymph nodes.

- Progression (PD): At least one of the following must be true:

  a. At least one new malignant lesion, which also includes any lymph node that was normal at baseline (< 1.0 cm short axis) and increased to ≥ 1.0 cm short axis during follow-up.

  b. Unequivocal progression of existing non-target lesions and non-target lymph nodes. (NOTE: Unequivocal progression should not normally trump target lesion and target lymph node status. It must be representative of overall disease status change.)

.

11.44 Overall Objective Status

The overall objective status for an evaluation is determined by combining the patient's status on target lesions, target lymph nodes, non-target lesions, non-target lymph nodes, and new disease as defined in the following tables:

For Patients with Measurable Disease

**Target Lesions & Target Lymph Nodes**	**Non-Target Lesions & Non-Target Lymph Nodes**	**New Sites of Disease**	**Overall Objective Status**
CR	CR	No	CR
CR	Non-CR/Non-PD	No	PR
PR	CR<br>Non-CR/Non-PD	No	PR
CR/PR	Not All Evaluated*	No	PR**
SD	CR<br>Non-CR/Non-PD<br>Not All Evaluated*	No	SD
Not all Evaluated	CR<br>Non-CR/Non-PD<br>Not All Evaluated*	No	Not Evaluated (NE)
PD	Unequivocal PD<br>CR<br>Non-CR/Non-PD<br>Not All Evaluated*	Yes or No	PD
CR/PR/SD/PD/Not all Evaluated	Unequivocal PD	Yes or No	PD
CR/PR/SD/PD/Not all Evaluated	CR<br>Non-CR/Non-PD<br>Not All Evaluated*	Yes	PD

*See Section 11.431
** NOTE: This study uses the protocol RECIST v1.1 template dated 2/16/2011. For data collection and analysis purposes the objective status changed from SD to PR in the ACCRU protocol RECIST v1.1 template as of 2/16/2011 and to match RECIST v1.1 requirements.

11.45 Symptomatic Deterioration: Patients with global deterioration of health status requiring discontinuation of treatment without objective evidence of disease progression at that time, and not either related to study treatment or other medical conditions, should be reported as PD due to "symptomatic deterioration." Every effort should be made to document the objective progression even after discontinuation of treatment due to symptomatic deterioration. A patient is classified as having PD due to "symptomatic deterioration" if any of the following occur that are not either related to study treatment or other medical conditions:

- Significant worsening of tumor-related symptoms as per investigator discretion.
- Decline in performance status to ≥ 3 on ECOG scale.

12.0 Descriptive Factors

12.1 ECOG PS: 0 vs. 1. vs. 2

12.2 Disease characteristics: Unresectable vs. Metastatic.

12.3 Prior treatment: TAS-102 vs. Regorafenib vs. neither vs both (TAS-102 and Regorafenib)

12.4 Primary tumor location: cecum vs. ascending vs. hepatic flexure vs. transverse vs. splenic flexure vs. descending vs. sigmoid vs. rectosigmoid vs. rectum vs other.

12.5 Microsatellite instability or mismatch repair deficiency tested: Yes vs. no.

12.51 If yes, MSI-High (MSI-H) vs. microsatellite stable/MSI-Low (MSS/MSI-L); loss of mismatch repair protein (MMR deficient, dMMR) vs. mismatch repair proteins intact (MMR proficient, pMMR).

12.6 KRAS (mutated vs. wildtype vs. unknown)

12.7 NRAS (mutated vs. wildtype vs. unknown)

12.8 BRAF V600E (mutated vs. wildtype vs. unknown)

13.0 Treatment/Follow–up Decision at Evaluation of Patient

13.1 Patients who have CR, PR, or SD will continue treatment per protocol up to 35 cycles. After 35 cycles, patients will go to event monitoring.

13.2 Patients who develop PD while receiving therapy will go to the event-monitoring phase.

13.3 Patients who go off protocol treatment for reasons other than PD or alternative therapy will go to the event-monitoring phase per Section 18.0.

13.4 Event monitoring is every 3 months for 3 years after registration. If patient is still alive 3 years after registration, no further follow up is required.

13.5 A patient is deemed *ineligible* if after registration, it is determined that at the time of registration, the patient did not satisfy each and every eligibility criteria for study entry. The patient may continue treatment off-protocol at the discretion of the physician as long as there are no safety concerns, and the patient was properly registered. The patient will go directly to the event-monitoring phase of the study (or off study, if applicable).

- If the patient received treatment, all data up until the point of confirmation of ineligibility must be submitted. Event monitoring will be required per Section 18.0 of the protocol.

- If the patient never received treatment, On-study material and the Off Treatment Form must be submitted. No further data submission is necessary.

13.6 A patient is deemed a *major violation*, if protocol requirements regarding treatment in cycle 1 of the initial therapy are severely violated that evaluability for primary end point is questionable. All data up until the point of confirmation of a major violation must be submitted. The patient will go directly to the event-monitoring phase of the study. The patient may continue treatment off-protocol at the discretion of the physician as long as there are no safety concerns, and the patient was properly registered. Event monitoring will be required per Section 18.0 of the protocol.

13.7 A patient is deemed a *cancel* if he/she is removed from the study for any reason before any study treatment is given. On-study material and the Off Treatment Form must be submitted. No further data submission is necessary.

13.8 Patients have the option to complete cfDNA testing at study completion and are encouraged to re-register with protocol ACCRU-GI-1611 (COLOMATE). The results of the cfDNA test will be used for eligible patients in protocol ACCRU-GI-1611 (COLOMATE) to identify genomic alterations and facilitate enrollment on COLOMATE companion clinical trials. Site will access results by creating an account at https://portal.guardanthealth.com. Sites will be notified by Guardant Health via email when results are available in the portal. See detailed instructions on the ACCRU website under Manuals and Forms.

14.0 Body Fluid Biospecimens

14.1 Summary Table of Research Blood/Blood Products to Be Collected for This Protocol

Indicate if specimen is mandatory or optional	Collection tube description and/or additive (color of tube top)	Volume to collect per tube (number of tubes to be collected)	Blood product being processed and submitted by participating site	Baseline[1]	Prior to dosing on Day 1 of Cycle 2	Restaging[2]	End of Treatment[3]	Additional processing required at site after blood draw?	Storage /shipping conditions[4]
Mandatory	EDTA K2 (Purple)	10 mL (1)	Whole Blood	X				No	Freeze /dry ice
Mandatory	EDTA K2 (Purple)	10 mL (4)	Platelet Poor Plasma and White blood cells	X	X	X	X	Yes	Freeze /dry ice
Optional	Streck tube[5]	10 mL (2)	Whole blood				X	No	Room temperature

1. May occur prior to treatment on Cycle 1 Day 1.
2. Day 1 of each cycle which is immediately following radiographic assessment for re-staging (day 1 of cycles 4,7,10 etc.)
3. Discontinuation of study treatment.

4. After all samples have been processed according to kit instructions, ship all specimens according to shipping instructions (see Section 14.2 for detailed shipping instructions.) Patients will complete cfDNA testing at study completion and then be encouraged to re-register with protocol ACCRU-GI-1611. See section 13.8

14.2 Kits are required for this study.

NOTE: You will be ordering and receiving kits from two different locations.

14.21 EDTA K2

14.211 Each kit will contain supplies and instructions for collecting, processing, and shipping specimens.

14.212 Participating institutions may obtain kits for **EDTA K2** by completing and faxing the Supply Order Form (found in the Forms Packet) to the number listed on the form. Fill out the site address to where the kits will be shipped on the Fax Supply form. A small but sufficient supply of the specimen collection kits should be ordered prior to patient entry. Unused/expired kits should be disposed of per institution policy. Do not send unused kits back to BAP. **Supply Order Forms must be filled in completely and legibly for quick processing.**

14.213 Kits will be sent via FedEx Ground at no cost to the participating institutions. **Allow up to two weeks to receive the kits.** Kits will arrive inside the shipping boxes.

14.214 Kits will not be sent via rush delivery service unless the participating institution provides their own FedEx account number or alternate billing number for express service. **ACCRU will not cover the cost for rush delivery of kits.**

14.22 Guardant 360 (Streck Tube)14.221 In order to receive kits and access to the Guardant portal, fill out the Guardant Site Setup form for each treating location and email to COLOMATEACCRU@mayo.edu.

14.222 If participating though ACCRU-GI-1611 (COLOMATE), sites will create an account at https://portal.guardanthealth.com. Once account is created, Guardant Health will ship the sites their kits and their own pre-printed TRFs (Test Requestion Form). Once that happens, kits and TRFs can be refreshed/replaced by contacting Guardant Health Client Services Team at 855-698-8887 or clientservices@guardanthealth.com

14.3 Shipping and Handling

NOTE: You will be shipping kits to two different locations.

14.31 EDTA K2

14.311 Verify ALL sections of the Blood Specimen Submission Form (see Forms Packet), BAP Requisition Form (provided in kit) and specimen collection labels are completed and filled in correctly.

14.312 Specimens must be shipped the same day they are drawn.

14.313 Ship tubes with a properly prepared cold pack. See kit instructions for specific details for cold pack preparation (i.e., frozen or refrigerated) and proper packing of blood and cold pack to avoid freezing of specimen.

14.314 Ship specimens via Priority Overnight service, Monday – Thursday, to BAP Freezer according to kit instructions. Do not send samples on weekends or just prior to federal holidays. If a patient can only be seen on Fridays, email the Biospecimen Manager (found on resource page) with the sample information and FedEx tracking number.

14.315 The BAP kits will include a smart shipper label (3x5 white barcoded label) affixed to the shipping boxes. The smart shipper label is a pre-addressed return label, which replaces the need for an air bill. Shipping costs will be covered by ACCRU if the shipping box provided with the BAP kit is used for shipping specimens to BAP Freezer.

14.316 BAP Freezer will receive the samples and immediately forward specimens to the ACCRU Research Base BAP Shared Resource, Stabile SL-16. At study completion, specimens will be forwarded to the Duke Phase I Biomarker Laboratory located at Duke University for future processing.

14.32 Guardant 360 (Streck Tube)

14.321 Complete the Test Requisition Form and barcode labels (provided in kit).

14.322 Ship tubes the same day as collecton with properly prepared gel pack. Do not freeze gel packs. Use as is.

14.323 Place the kit into the preprinted FedEx Clinical Pak and call FedEx for a pickup to be shipped to Guardant Health.

14.324 Detailed Blood Draw and Shipping Instructions are located on the ACCRU website under Manuals and Forms.

14.4 Study Methodology and Storage Information

14.41 Blood/blood product samples will be collected for the following research:

14.411 Whole blood for pharmacogenomics

Whole blood will be stored for future pharmacogenetic assays (e.g., genetic polymorphisms such as those known to regulate angiogenesis, inflammation, immunity, auto-immunity, and antibody or drug action of clearance) that may correlate with efficacy and tolerability. The Duke Phase I Biomarker Laboratory located at Duke University may analyze the DNA for the presence of markers of interest using standard laboratory protocols.

14.412 Soluble protein (blood-based) biomarkers

Blood (platelet poor plasma, and white blood cells (buffy coat)) will be collected at baseline, every restaging, and progression for future analysis. The Duke Phase I Biomarker Laboratory located at Duke University may analyze for the following, but are not limited to soluble HGF, c-MET, EGF, HBEGF, HER1-3, FGFa, FGFb, VEGFA-D, PlGF, VEGFR2, GAS6, AXL, SDF1, Ang2, and TIE-2. Additional biomarkers may also be explored using multiplex array technology. Final biomarker selection will reflect the best science at the time of analysis.

14.413 Plasma for mutational analysis

Blood (platelet poor plasma) will be stored for future analysis of cell free DNA (cfDNA) and will be collected at baseline, every restaging, and at the 30-day post-treatment visit. The Duke Phase I Biomarker Laboratory located at Duke University may analyze for the following, but are not limited to *HER2* amplification, *EGFR* amplification, BRAF mutations, and extended KRAS/NRAS testing (exons 2, 3, and 4). Final biomarker selection will reflect the best science at the time of analysis.

14.414 Future use of patient samples

Any remaining biological materials (platelet poor plasma, and buffy coats) at the end of the study will be retained for possible use in future biomarker research.

14.5 Return of Genetic Testing Research Results

Results of CLIA-certified assays (e.g., Guardant360) are permitted to be shared with patients and their treating physicians. Because the results generated by other genetic testing included in this section are not currently anticipated to have clinical relevance to the patient or their family members, the genetic results will not be disclosed to the patients or their physicians.

If at any time, genetic results are obtained that may have clinical relevance, IRB review and approval will be sought regarding the most appropriate manner of disclosure and whether or not validation in a CLIA-certified setting will be required. Sharing of research data with individual patients should only occur when data have been validated by multiple studies and testing has been done in CLIA-approved laboratories.

15.0 Drug Information

IND number (144530)

- Investigator brochure available on ACCRU website.

15.1 **Pemigatinib (INCB054828):**

15.11 **Background:** Pemigatinib is an inhibitor of the FGFR family of receptor tyrosine kinases that is proposed for the treatment of malignant diseases or other diseases related to FGFR dysregulation. Aberrant signaling through FGFR resulting from gene amplification or mutation, chromosomal translocation, and ligand-dependent activation of the receptors has been demonstrated in multiple types of human cancers.

15.12 **Formulation**: Pemigatinib drug substance is a white to off-white to light brown to yellow solid. The pemigatinib drug product is formulated as immediate release tablets in strengths of 0.5, 2, 4.5, 9 and 13.5 mg. The tablets of all strengths contain the active drug substance along with the excipients microcrystalline cellulose, sodium starch glycolate, and magnesium stearate.

15.13 **Preparation and storage**: Pemigatinib should be stored at room temperature between 15°-30°C (59°-86°F) as detailed on the investigational product labeling.

15.14 **Administration:** The effect of food on pemigatinib exposure is modest and is not expected to be clinically significant; as a result, pemigatinib may be administered orally either with or without food.

15.15 **Pharmacokinetic information**:
Time to peak, serum: 0.5 - 6 hours
Clearance: 11.2 L/hour
Distribution: Volume of distribution – 151 L
Protein Binding: 90.6%
Metabolism: In vitro studies suggested that pemigatinib is predominantly metabolized by CYP3A4.
Pemigatinib is not a potent inhibitor or inducer of the major CYPs evaluated.
Half-life elimination (mean): 15 hours

15.16 **Potential Drug Interactions**:
The data from Study INCB 54828-104 demonstrated a clinically significant effect on pemigatinib exposure when a potent CYP3A4 inhibitor (itraconazole) or inducer (rifampin) is coadministered, and physiologically based pharmacokinetic modeling showed that coadministration of a moderate CYP3A4 inducer decreased the pemigatinib AUC by at least 50%. In addition, the data from Study INCB 54828-106 demonstrated a modest effect on the overall exposure of pemigatinib following coadministration of the gastric pH-modifying agents esomeprazole and ranitidine. Therefore, pemigatinib can be dosed without respect to concomitant use of PPIs or H2 antagonists.
In vitro transport studies indicated that pemigatinib is a substrate of both P-gp and BCRP, but it is unlikely that efflux by these 2 transporters plays an important role in the oral absorption of
pemigatinib. Pemigatinib is an inhibitor of P-gp, OCT2, and MATE1. However, at the proposed therapeutic dose, physiologically based pharmacokinetic

modeling showed that a clinical drug-drug interaction is unlikely to occur as a result of
pemigatinib-mediated inhibition of these transporters.

Refer to the study Protocols for details regarding restricted and prohibited concomitant medications.

15.17 **Known potentialAdverse Events**:
Summary of Treatment-Emergent Adverse Events Reported in ≥ 10% of Participants in Study INCB 54828-101: Very common adverse events (≥ 10%): Hyperphosphatemia, hypophosphatemia, hyponatremia, dry eye, nausea, stomatitis, diarrhea, constipation, dry mouth, palmar-plantar erythrodysesthesia syndrome, alopecia, dry skin, arthralgia, fatigue, and blood creatinine increased

Common adverse events (1-10%): Serous retinal detachment, retinal detachment, detachment of retinal pigmented epithelium, subretinal fluid, punctate keratitis, vision blurred, trichiasis, nail toxicity, nail disorder, nail discoloration, nail dystrophy, nail ridging, nail infection, onychomycosis, onychalgia, onychoclasis, onycholysis, onychomadesis, and paronychia

Less common adverse events (<1%): Nail hypertrophy, retinal thickening, chorioretinal folds, chorioretinal scar, maculopathy, hair growth abnormal, and photopsia

Summary of Treatment-Emergent Adverse Events Reported in ≥ 10% of Participants in Study INCB 54828-201:
Diarrhea, alopecia, stomatitis, fatigue, constipation, dry mouth, hyperphosphatemia, dysgeusia, decreased appetite, nausea, asthenia, urinary tract infection, abdominal pain, vomiting, anemia, back pain, dry eye, dry skin, blood creatinine increased, pyrexia, arthralgia, hematuria, weight decreased, dizziness, acute kidney injury, cough, hyponatremia
Summary of Treatment-Emergent Adverse Events Reported in ≥ 10% of Participants in Study INCB 54828-202:
Hyperphosphatemia, alopecia, diarrhea, fatigue, dysgeusia, dry mouth, stomatitis, decreased appetite, nausea, constipation, arthralgia, hyperphosphatemia, vomiting, back pain, dry eye, dry skin, abdominal pain, oedema peripheral, anemia, hypercalcemia, pain in extremity, urinary tract infection, weight decreased, headache, asthenia, dehydration, palmar-plantar erthrodysesthesia syndrome, gastrointestinal reflux disease, myalgia.

15.18 **Drug procurement:**
Incyte will supply Pemigatinib through Catalent. Each participating ACCRU treating location will order the drug from Catalnet using the "Investigational supplies shipment and receipt verification form" found on the ACCRU web site. This form will be emailed to:

Email: Philadelphia.distributionrequests@catalent.com

Each participating ACCRU treating location will be responsible for monitoring the supply of pemigatinib and will use the "Investigational supplies shipment and

receipt verification form" to order additional supplies as needed.

Outdated or remaining drug is to be destroyed on-site as per procedures in place at each institution.

15.19a Temperature excursions that occur at the site should be reported by the site using the Temperature Excursion Report Form found on the ACCRU web site for this study and emailed to: tempex@incyte.com

15.19b **Nursing Guidelines:**

15.19b1 Due to the early investigational nature of this agent, not all side effects can be known at this time. Monitor patients closely and report any side effects to the treating provider/study team.

15.19b2 Gastrointestinal side effects can be seen including nausea, vomiting, constipation, and diarrhea. Treat symptomatically and monitor for effectiveness.

15.19b3 Monitor LFTs. Report elevated LFTs to the treating provider.

15.19b4 Patients may have visual changes, including blurred vision and dry eyes. Instruct patients to report any dry eyes, pain and/or visual changes to the study team immediately.

15.19b5 Patients may report fatigue, monitor hemoglobin as anemia can be seen. Instruct patients on energy conserving lifestyle.

15.19b6 Pemigatinib may be administered with or without food.

15.19b7 Electrolyte abnormalities can be seen. Monitor lab values and report abnormal lab values to treating provider.

15.19b8 Severe and serious ocular conditions, including retinal detachment have been seen. Instruct patient to report any visual changes to the study team.

15.19b9 Nail changes can be seen, additionally patients may experience palmar-planta erythrodysthesia has been documented. Instruct patients to report nail and or skin changes to the study team.

16.0 Statistical Considerations and Methodology

16.1 Overview and Study Design:

This is a single-arm Phase II study which is designed to assess initial evidence of efficacy

of Pemigatinib in patients with metastatic colorectal cancer with activating FGFR alterations. Overall response rate (ORR) will be used as the primary endpoint. Based on historical data and clinical experience, an ORR of 5% or less would not be of interest (null hypothesis), and an ORR of 20% or more would be considered of interest. To assess this endpoint, a Simon two-stage minimax design will be used. In addition to ORR, clinical benefit rate (complete response, partial response, or stable disease), progression-free survival, overall survival, and adverse event rates will be of specific interest. As correlative research, we will assess plasma pharmacodynamics biomarkers or response and resistance to therapy.

Given an estimated prevalence of 5% for eligible activating FGFR alterations in the proposed population, approximately 500 metastatic colorectal cancer patients will need to be screened to identify 24 genomically eligible patients for this study.

Patients will be evaluated for response to therapy every 9 weeks with computed tomography (CT) or magnetic resonance imaging (MRI). Response and progression will be evaluated using RECIST 1.1 (confirmation scan will not be required). Additionally, blood samples will be drawn at baseline, cycle 2 day 1, day 1 following each restaging scan, and end of treatment for analysis of cfDNA.

16.11 Primary Endpoint

The primary endpoint for this study is overall response rate (ORR) at 36 weeks after registration, which is defined as the percentage of patients, among evaluable patients, who experience an objective response within 36 weeks. Objective response is defined as a complete or partial response per RECIST 1.1, without the confirmation scan. The evaluable patients are those who are eligible, consented, received any protocol treatment, and are not major violations.

16.2 Statistical Design

16.21 Decision Rule

A Simon two-stage minimax design will be used. The largest success proportion where the proposed treatment regimen would be considered ineffective in this population is 5%, and the smallest success proportion that would warrant subsequent studies with the proposed regimen in this patient population is 20%. The following two-stage design uses 12 or 21 evaluable patients to test the null hypothesis that the true success proportion in this given patient population is at most 5%.

NOTE: We consider a success to be a patient with an objective response by 36 weeks post-registration (i.e. complete response or partial response).

16.211 STAGE 1:

Enter 12 evaluable patients into the study. If 0 successes are observed in the first 12 evaluable patients, we will consider this treatment ineffective in this patient population and terminate this study. Otherwise, if at least 1 success is observed, we will proceed to Stage 2.

16.212 STAGE 2:

Enter an additional 9 evaluable patients into the study. If 2 or fewer successes are observed in the first 21 evaluable patients, we will consider this treatment ineffective in this patient population. If 3 or more successes are observed in the first 21 evaluable patients, we may recommend further testing of this regimen in subsequent studies in this population.

16.213 Over Accrual

If more than the target number of patients are accrued, the additional patients will not be used to evaluate the stopping rule or used in any decision making process.

16.214 NOTE:

We will not suspend accrual between stages to allow the first 12 patients to become evaluable, unless undue toxicity is observed.

16.22 Sample Size

The two-stage study design to be utilized is fully described in section 16.21. A minimum of 12 and a maximum of 21 evaluable patients will be accrued onto this study unless undue toxicity is encountered. We anticipate accruing an additional 3 patients to account for ineligibility, cancellation, major treatment violations, or other reasons. Maximum projected accrual is therefore 24 patients. All sample size calculations were computed using PASS 15 software.

16.23 Accrual Time and Study Duration

The anticipated accrual rate is 1 patient per month. Therefore, the accrual period for the first stage of this study is expected to be approximately 12 months. If patients are accrued to the second stage, the total accrual period is expected to be approximately 21 months. Should the study continue to full accrual, the final analysis can begin at most 30 months after the trial begins; as soon as the last patient has either responded to therapy, or been observed for 36 weeks.

16.24 Power and Significance Level

We assume that the number of successes is binomially distributed, our null success proportion is 0.05, and our alternative success proportion is .20. Given these, along with an assumed type I error rate of 0.1, a sample size of 21 evaluable patients will provide 82% exact power (exact type II error rate = 0.18) to detect a true ORR of 20% or greater (vs. the null hypothesis that the true ORR is at most 5%).

16.25 Operating Characteristics

The probability of declaring that this regimen warrants further studies (i.e. statistical power) under various success proportions and the probability of

stopping accrual after the first stage can be tabulated as a function of the true success proportion as shown in the following table.

If the true success proportion is:	0.05	0.1	0.15	0.2
then the probability of declaring that the regimen warrants further studies is:	0.08	0.35	0.63	0.82
and the probability of stopping at stage 1 is:	0.54	0.28	0.14	0.07

16.25 Other Considerations

Adverse events, quality/duration of response, and patterns of treatment failure observed in this study, as well as scientific discoveries or changes in standard of care will be taken into account in any decision to terminate the study.

16.3 Analysis Plan

The analysis for this trial will begin at planned time points (see 16.2) and at the time the patients have become evaluable for the primary endpoint. Such a decision will be made by the Statistician and Study Chair, in accord with CCS Standard Operating Procedures, availability of data for secondary endpoints (e.g. laboratory correlates), and the level of data maturity.

16.31 Primary Objective

16.311 Definition

The primary endpoint for this study is overall response rate (ORR) at 36 weeks after registration, which is defined as the percentage of patients, among evaluable patients, who experience an objective response. Objective response is defined as a complete or partial response per RECIST v1.1, without the confirmation scan. The evaluable patients are those who are eligible, consented, received any protocol treatment, and are not major violations.

16.312 Estimation

The proportion of successes will be estimated by the number of successes divided by the total number of evaluable patients. Confidence intervals for the true success proportion will be calculated according to the approach of Clopper and Pearson.

16.313 Over Accrual

If more than the target number of patients are accrued, the additional patients will not be used to evaluate the stopping rule or used in any decision making process. However, they will be included in final point estimates and confidence intervals as though they were accrued in the final stage.

16.32 Secondary Objectives

The following objectives will be evaluated: clinical benefit rate, progression-free survival, overall survival, and adverse event rates. Historical controls will be used for comparisons when appropriate.

16.321 Clinical Benefit Rate

Clinical Benefit Rate is defined as the number of patients that experience a complete or partial response within 36 weeks post registration, or have stable disease for at least 36 weeks post registration, divided by the number of evaluable patients. Analysis of this endpoint will mirror that of the primary objective (section 16.31).

16.322 Progression-free Survival

Progression-free survival (PFS) is defined as the time from study entry to the first of either disease progression or death from any cause, where disease progression is determined based on RECIST 1.1 criteria. PFS will be estimated using the Kaplan-Meier method. The median PFS and corresponding 95% confidence interval will be reported. Patients who do not experience disease progression or death while on protocol will be censored at the last disease assessment date.

16.323 Overall survival

Overall survival (OS) is defined as the time from study entry to death from any cause. Analysis of this endpoint will mirror that of PFS (section 16.322).

16.324 Patient-reported Outcomes

Patient reported quality of life (QOL) outcomes will be collected using the Linear Analog Self-Assessment (LASA) Questionnaire. Data will be collected each cycle. Mean values of the first question (regarding overall QOL) at each cycle will be plotted. Additional analyses using data collected from the LASA questionnaire may be performed but will be considered exploratory.

16.325 Adverse Event Rates

All patients who have initiated treatment will be considered evaluable for adverse event analyses. Adverse events will be summarized by frequency and severity using CTCAE v 5.0. They will be reviewed to determine overall adverse event patterns, and will be closely monitored throughout the study.

16.33 Correlative Research

16.331 Plasma Biomarkers

In the assessment of plasma pharmacodynamic biomarkers of response

and resistance to therapy, descriptive statistics (e.g. means, standard deviations, 95% confidence intervals for continuous variables and frequencies for discrete data) and graphical analyses will be used for all correlative laboratory procedures. The associations between laboratory parameters and clinical response will be evaluated using the two-sample t-test or Fisher's exact test, as appropriate. Response rate with the corresponding 95% exact binomial confidence interval will be provided.

16.4 Data & Safety Monitoring

16.41 Review

The study chair(s) and study statisticians will review the study at least twice per year to identify accrual, adverse event, and any endpoint problems that might be developing. The Mayo Clinic Cancer Center (MCCC) Data Safety Monitoring Board (DSMB) is responsible for reviewing accrual and safety data for this trial at least twice per year, based on reports provided by the study statistician. Any safety issues requiring protocol changes are communicated through protocol amendments.

16.42 Adverse Event Stopping Rules

The monitoring rule specified below is based on the knowledge available at study development. We note that the Adverse Event Monitoring Rule may be adjusted in the event of either (1) the study re-opening to accrual after any temporary suspension or (2) at any time during the conduct of the trial and in consideration of newly acquired information regarding the adverse event profile of the treatment(s) under investigation. The study team may also choose to suspend accrual because of unexpected adverse event profiles that have not crossed the specified rule below.

Accrual may be temporarily suspended to this study if at any time we observe events considered at least probably related to study treatment (i.e., an adverse event with attribute specified as "probable" or "definite") that satisfy any of the following:

- If 3 or more patients in the first 9 treated patients (or 35% or more of all patients after 9 are treated) experience a grade 4 or 5 adverse event at least probably related to treatment.

We note that we will review all Grade 4 and 5 adverse events to verify their attribution and to monitor the emergence of a previously unrecognized treatment-related adverse event.

17.0 Pathology Considerations/Tissue Biospecimens

17.1 Tissue Biospecimen Submission

17.11 Summary Table of Tissue Biospecimens for This Protocol

Type of tissue biospecimen to submit	Mandatory or optional	When to submit	Reason for submission (background/methodology section)	Where to find specific details for biospecimen submission
FFPE tissue block with corresponding H&E slide or up to twenty-five (25) 4-micron, unstained slides and up to three (3) corresponding H&E slides from **primary tumors present prior to study entry** (if primary tissue not available, please use tissue from metastatic lesions)	Mandatory	Within 90 days from randomization	Correlative studies (Section 17.3)	Section 17.2

17.2 Paraffin Embedded Tissue Blocks/Slides

17.21 Submit one formalin fixed paraffin-embedded (FFPE) tumor tissue block with largest amount of invasive tumor (at least 1 cm of tumor for cases of surgical resection) from original surgery at the time of diagnosis. Biopsy material obtained at the time of metastatic diagnosis may be submitted, if blocks from the surgical resection are inadequate or unavailable **a corresponding H&E slide for each submitted block must be provided**. The H&E slide for each block should be reviewed by the institution's pathologist to assess tissue quality prior to submission.

17.22 The FFPE tissue block is preferred; however, **if an institution is unable to provide a tissue block**, cut up to 25 (twenty-five) 4-micron sections and mount on charged glass slides. **Label the slides with ACCRU patient ID number, accession number, and order of sections, and thickness of sections. NOTE:** do <u>not</u> place "sticky" labels directly on slides, the institution label needs to be visible.. H&E stain every tenth slide (i.e., slides labeled 1, 11, 21, etc.). The H&E slides should be reviewed by the institution's pathologist to assess tissue quality prior to submission. For samples containing less than 7 square millimeters of tumor tissue, multiple sections should be mounted onto each slide to ensure that the appropriate amount of tumor tissue is available. Ideally, each slide should have a minimum of 75% tumor tissue on the slide to be deemed adequate for study. **Do not bake or place covers slips on the slides.**

17.23 The following materials below are mandatory (unless indicated otherwise) and required for shipment:

- Paraffin embedded tissue blocks with corresponding H&E slide(s) (OR up to 25 (twenty-five) unstained slides with corresponding H&E slide(s)).
- Specimen Submission: Tissue form
- Surgical Pathology Report
- Operative Report *(optional)*

NOTE: Please include the ACCRU patient ID number on all materials listed above.

17.24 The block/slides must be appropriately packed to prevent damage (e.g., slides should be placed in appropriate slide container) and placed in an individual plastic bag. Label the bag with the protocol number, ACCRU patient ID number, and patient initials.

17.25 Tissue specimens must be shipped ≤90 days from date of registration.

17.26 Verify that the appropriate sections of the Specimen Submission: Tissue form are completed and filled in correctly. Enter information from the Specimen Submission: Tissue form into the remote data entry system on the same day the specimen is submitted (see Forms Packet).

17.27 Ship all block/slide tissue specimens and accompanying materials to the ACCRU Research Base:

ACCRU Operations Office
Attn: PC Office (ACCRU-GI-1701)
RO_FF_03_24-CC/NW Clinic
200 First Street SW
Rochester, MN 55905

17.28 When an appropriate request is submitted, the ACCRU Operations Office will forward the block(s) to the ACCRU Research Base PRC, 2915 Valley high Drive, Mayo Clinic Rochester (Attn: PRC Supervisor). At study completion specimens will be sent to the Duke Phase I Biomarker Laboratory located at Duke University for processing as outlined in Section 17.3.

17. 3 Study Methodology and Storage Information

Submitted tissue samples will be analyzed as follows:

17.31 At the completion of the study, any unused/remaining material will be stored in Duke Phase I Biomarker Laboratory located at Duke University for future research according to the patient consent permission (see Section 6. 5). Potential future research may include immunohistochemistry (IHC) analyses to analyze predictive biomarkers, changes in expression pattern with therapy, and correlation with response and/or adverse events. When a protocol is developed, it will be presented for IRB review and approval.

17.32 Banking of tumor tissue, according to the patient consent permission (see Section 6. 5), is for future research. As protocols are developed, they will be presented for ACCRU and IRB review and approval. (This collection is part of a general strategy of investigation for ACCRU studies).

17.33 The institutional pathologist will be notified by the Pathology Coordinator if the block may be depleted.

17.34 Blocks requested to accommodate individual patient management will be returned promptly upon request.

17.35 Return of Genetic Testing Research Results: No genetic specimens will be collected from tissue biospecimens for this study. If future genetic testing is being requested for stored tissue, patient reconsent is required.

18.0 Records and Data Collection Procedures

All data must be entered by Remote Date Entry (RDE) and completed by qualified and authorized personnel. Access the RAVE RDE system through the iMedidata portal at https://login.imedidata.com. All data on the CRF must reflect the corresponding source document. Please refer to the ACCRU website for instructions (https://www.accru.org/).

18.1 Submission Timetable

Initial Material(s)

**CRF**	**Active-Monitoring Phase**<br>(Compliance with Test Schedule Section 4.0)
Institutional Contacts	≤2 weeks after registration
On-Study	
On-Study: Prior Surgery[1]	
On-Study: Prior Systemic Therapy	
On-Study: Prior Radiation[1]	
Adverse Event: Baseline	
RECIST Measurements: Baseline	
Laboratory Tests & Results: Baseline	
Supporting Documentation: Baseline[2]	
Specimen Submission: Blood (Baseline) (see Section 14.0)	
Specimen Submission: Tissue (Baseline) (see Section 17.0)	
Patient Status: Baseline	
Notice of New Primary[1]	
OP and Path Reports (see Section 17.0)[2]	
Imaging Report[2]	
Patient Questionnaire Booklet-PRO/QOL: Linear Analogue Self Assessment (LASA)	≤ 2 weeks after registration - Patient questionnaire booklet must be used; copies are not acceptable for this submission.
PRO/QOL : Booklet Compliance	≤ 2 weeks after registration - This form must be completed

CRF	Active-Monitoring Phase (Compliance with Test Schedule Section 4.0)
	only if the (LASA) contains absolutely NO patient provided assessment information.
Off Treatment	Submit ≤2 weeks after registration if withdrawal/refusal occurs prior to beginning protocol therapy
ACCRU Deviation Form[1]	Submit only if applicable during all phases of the study (initial, active and observation)

1. Submit only if applicable.
2. Upload via the Supporting Documentation: Baseline form. This is in addition to the pathology material requirements for tissue submission (Section 17.0). Required items to be uploaded include: COLOMATE Companion Trial Recommendation Form (Only required if participating on ACCRU-GI-1611), Imaging Report, and Pathology report.

Test Schedule Material(s)

CRF	Active-Monitoring Phase (Compliance with Test Schedule Section 4.0)	
	At each evaluation during treatment	At end of treatment
Treatment (Intervention)	X	X
Treatment (Intervention): Dose Modifications, Omissions and Delays[1]	X	
Adverse Events: Solicited	X	X
Adverse Events: Other[1]	X	X
RECIST Measurements[1,2]	X	X
Supporting Documentation[1,2]	X	X
Laboratory Tests and Results	X	X
Specimen Submission: Blood (see Section 14.0)[1]	X	
Patient Status: Treatment (Intervention)	X	X
PRO/QOL: Booklet Compliance	X	X
Patient Questionnaire Booklet-PRO/QOL: Linear Analogue Self Assessment (LASA) (if applicable)[3]	X	X
Patient Medication Diary[5]	X	
PRO/QOL : Booklet Compliance (if applicable)[4]	X	X

CRF	Active-Monitoring Phase (Compliance with Test Schedule Section 4.0)	
	At each evaluation during treatment	At end of treatment
Notice of New Primary[1]	X	X
Consent Withdrawal (choose appropriate form)[1]<br>• Consent Withdrawal: Specimen Only<br>•<br>• Consent Withdrawal: All Follow-Up	X	X
Off Treatment		X
ACCRU Deviation Form[1]	X	X

1. Submit only if applicable.
2. Upload imaging for all scans to support RECIST measurements and clinic note in the Supporting Documentation Form.
3. Patient questionnaire booklet must be used; copies are not acceptable for this submission.
4. This form must be completed only if the Linear Analogue Self Assessment (LASA) contains absolutely NO patient provided assessment information.
5. This form is to be completed by the patient and uploaded to RAVE in the Treatment (Intervention) folder for each cycle.

Follow-up Material(s)

CRF	Event Monitoring Phase[1]				
	q. 3 months until PD	At PD	After PD q. 3 mos.	Death	New Primary
Patient Status: Survival and Disease Status Follow-Up/Event Monitoring	X[2]	X[2]	X	X	At each occurrence
Adverse Events: Late[3]					X
Supporting Documentation[3]					
Notice of New Primary[3]					X
Consent Withdrawal (choose appropriate form)[3]<br>• Consent Withdrawal: Specimen Only					X

CRF	Event Monitoring Phase[1]				
	q. 3 months until PD	At PD	After PD q. 3 mos.	Death	New Primary
• Consent Withdrawal: All Follow-Up					
Lost to Follow-Up[4]					

1. If a patient is still alive 3 years after registration, no further follow-up is required.
2. Upload a copy of documentation of response or progression in RAVE on the Supporting Documentation Form.
3. Submit only if applicable.
4. Patients are eligible to be confirmed lost to follow-up after 2 years of unsuccessful contact with the patient.

19.0 Budget

19.1 Each site should review the test schedule (Section 4.0), taking into account local and regional coverage policies, to determine which items are standard of care and which are research at their site. Refer to the payment synopsis for funding provided per accrual for covering study costs, as well as any additional invoiceables that may be allowed.

19.2 Other budget concerns:

19.21 Pemigatinib will be supplied by Incyte.

20.0 References

Abdel-Rahman O. Targeting FGF receptors in colorectal cancer: from bench side to bed side. *Future Oncol* 2015; 11(9): 1373-1379.

Almodovar K, Iams WT, Meador CB, et al: Longitudinal Cell-Free DNA Analysis in Patients with Small Cell Lung Cancer Reveals Dynamic Insights into Treatment Efficacy and Disease Relapse. J Thorac Oncol 13:112-123, 2018.

Berger G, Hanahan D. Modes of resistance to anti-angiogenic therapy. *Nat Rev Cancer* 2008; 8:592-603.

Brooks AN, Kilgour E, Smith PD. Molecular pathways: fibroblast growth factor signaling: a new therapeutic opportunity in cancer. *Clin Cancer Res* 2012; 18(7):1855-62.

Casanovas O, Hicklin DJ, Bergers G, Hanahan D. Drug resistance by evasion of antiangiogenic targeting of VEGF signaling in late-stage pancreatic islet tumors. *Cancer Cell* 2005; 8(4):299-309.

Corcoran RB, Andre T, Atreya CE, et al: Combined BRAF, EGFR, and MEK Inhibition in Patients with BRAFV600E-Mutant Colorectal Cancer. Cancer Discov, 2018.

Clinical Trial Facilitation Group (CTFG). Recommendations related to contraception and pregnancy testing in clinical trials. September 15, 2014. http://www.hma.eu/ctfg.html. Accessed April 04, 2018.

Dailey L, Ambrosetti D, Mansukhani A, Basilico C. Mechanisms underlying differential responses to FGF signaling. Cytokine Growth Factor Rev 2005;16:233-247.

Diaz LA, Jr., Williams RT, Wu J, et al: The molecular evolution of acquired resistance to targeted EGFR blockade in colorectal cancers. Nature 486:537-40, 2012.

Dienstmann R, Rodon J, Prat A, et al. Genomic aberrations in the FGFR pathway: opportunities for targeted therapies in solid tumors. *Ann Oncol* 2014; 25(3):552-63.

Donnem T, Al-Shibli K, Al-Saad S, Busund LT, Bremnes RM. Prognostic impact of fibroblast growth factor 2 in non-small cell lung cancer: coexpression with VEGFR-3 and PDGF-B predicts poor survival. J Thorac Oncol 2009;4:578-585.

Eisenhauer EA, Therasse P, Bogaert J, Schwartz LH, Sargent D, Ford R, Dancey J, Arbuck S, Gwyther S, Mooney M, Rubinstein L, Shankar L, Dodd L, Kaplan R, Lacombe D, Verweij J. New response evaluation criteria in solid tumors: revised RECIST guideline (version 1.1). Eur J Cancer 45(2): 228-247, 2009.

Eswarakumar VP, Lax I, Schlessinger J. Cellular signaling by fibroblast growth factor receptors. Cytokine Growth Factor Rev 2005;16:139-149.

Folprecht G, Beer P, Salazar R, et al. Frequency of potentially actionable genetic alterations in EORTC SPECTAcolor. *Ann Oncol* 2016; 27(suppl 6): 458O.

Fuks Z, Persaud RS, Alfieri A, et al. Basic fibroblast growth factor protects endothelial cells against radiation-induced programmed cell death in vitro and in vivo. Cancer Res 1994;54:2582-2590.

Graham RP, Barr Fritcher EG, Pestova E, et al: Fibroblast growth factor receptor 2 translocations in intrahepatic cholangiocarcinoma. Hum Pathol 45:1630-8, 2014.

Gill S, Blackstock AW, Goldberg RM: Colorectal cancer. Mayo Clin Proc 82:114-29, 2007.

Goke F, Goke A, von Massenhausen A, et al. Fibroblast growth factor receptor 1 as a putative therapy target in colorectal cancer. *Digestion* 2013; 88(3):172-81.

Greulich H, Pollock PM. Targeting mutant fibroblast growth factor receptors in cancer. *Trends Mol Med* 2011; 17:283-292.

Guagnano V, Kauffmann A, Wöhrle S, et al. FGFR genetic alterations predict for sensitivity to NVP-BGJ398, a selective pan-FGFR inhibitor. Cancer Discov 2012;2:1118-1133.

Investigator's Brochure: INCB 54828-101. Version 2, September 2016.

Kilgour E, Ferry D, Saggese M, et al. Exploratory biomarker analysis of a phase I study of AZD4547, an inhibitor of fibroblast growth factor receptor (FGFR), in patients with advanced solid tumors. J Clin Oncol 2014;32(suppl): Abstract 11010.

Knights V, Cook SJ. De-regulated FGF receptors as therapeutic targets in cancer. Pharmacol Ther 2010;125:105-117.

Kunii K, Davis L, Gorenstein J, et al. FGFR2-amplified gastric cancer cell lines require FGFR2 and Erbb3 signaling for growth and survival. Cancer Res 2008;68:2340-2348.

Le DT, Uram JN, Wang H, et al: PD-1 Blockade in Tumors with Mismatch-Repair Deficiency. N Engl J Med 372:2509-20, 2015.

Marek L, Ware KE, Fritzsche A, Hercule P, et al. Fibroblast growth factor and FGF receptor-mediated autocrine signaling in non-small-cell lung cancer. *Mol Pharmacol* 2009; 75(1): 196-207.

Mathur A, Ware C, Davis L, et al. FGFR2 is amplified in the NCI-H716 colorectal cancer cell line and is required for growth and survival. *PLoS One* 2014; 9(6):e98515.

Montagut C, Argiles G, Ciardiello F, et al: Efficacy of Sym004 in Patients With Metastatic Colorectal Cancer With Acquired Resistance to Anti-EGFR Therapy and Molecularly Selected by Circulating Tumor DNA Analyses: A Phase 2 Randomized Clinical Trial. JAMA Oncol:e175245, 2018.

Nogova L, Sequist LV, Garcia JMP, et al. Evaluation of BGJ398, a fibroblast growth factor receptor1-3 kinase inhibitor, in patients with advanced solid tumors harboring genetic alterations in fibroblast growth factor receptors: results of a global phase I, dose-escalation and dose- expansion study. J Clin Oncol 2016;35:157-165.

Pardo OE, Arcaro A, Salerno G, Raguz S, Downward J, Seckl MJ. Fibroblast growth factor-2 induces translational regulation of Bcl-XL and Bcl-2 via a MEK-dependent pathway: correlation with resistance to etoposide-induced apoptosis. J Biol Chem 2002;277:12040-12046.

Qing J, Du X, Chen Y, et al. Antibody-based targeting of FGFR3 in bladder carcinoma and t(4;14)-positive multiple myeloma in mice. J Clin Invest 2009;119:1216-1229. Lamont FR, Tomlinson DC, Cooper PA, Shnyder SD, Chester JD, Knowles MA. Small molecule FGF receptor inhibitors block FGFR-dependent urothelial carcinoma growth in vitro and in vivo. Br J Cancer 2011;104:75-82.

Rades D, Setter C, Dahl O, Schild SE, Noack F. Fibroblast growth factor 2 – a predictor of outcome for patients irradiated for stage II-III non-small cell lung cancer. Int J Radiat Oncol Biol Phys 2012;82:442-447.

Sequist LV, Cassier P, Varga A, et al. Phase I study of BGJ398, a selective pan-FGFR inhibitor in genetically preselected advanced solid tumors. In: Proceedings of the 105th Annual Meeting of the American Association for Cancer Research, April 5-9, 2014, San Diego, CA. Cancer Res 2014;74(19 suppl): Abstract CT326.

Siegel RL, Miller KD, Jemal A: Cancer statistics, 2018. CA Cancer J Clin 68:7-30, 2018

Sharpe R, Pearson A, Herrera-Abreu MT, et al. FGFR signaling promotes the growth of triple-negative and basal-like breast cancer cell lines both in vitro and in vivo. Clin Cancer Res 2011;17:5275-86.

Stransky N, Cerami E, Schalm S, et al. The landscape of kinase fusions in cancer. *Nat Commun* 2014; 5:4846.

Strickler JH, Loree JM, Ahronian LG, et al: Genomic Landscape of Cell-Free DNA in Patients with Colorectal Cancer. Cancer Discov 8:164-173, 2018.

Tabernero J, Bahleda R, Dienstmann R, et al. Phase I dose-escalation study of JNJ-42756493, an oral pan-fibroblast growth factor receptor inhibitor, in patients with advanced solid tumors. J Clin Oncol 2015;33:3401-3408.

Terai H, Soejima K, Yasuda H, et al. Activation of the FGF2-FGFR1 autocrine pathway: a novel mechanism of acquired resistance to gefitinib in NSCLC. Mol Cancer Res 2013;11:759-767.

Turner N, Pearson A, Sharpe R, et al. FGFR1 amplification drives endocrine therapy resistance and is a therapeutic target in breast cancer. Cancer Res 2010;70:2085-2094.

Weiss J, Sos ML, Seidel D, et al. Frequent and focal FGFR1 amplification associates with therapeutically tractable FGFR1 dependency in squamous cell lung cancer. Sci Transl Med 2010;2:62ra93.

Wesche J, Haglund K, Haugsten EM. Fibroblast growth factors and their receptors in cancer. *Biochem J* 2011; 437:199-21

Appendix I: Acceptable Contraception

A woman of childbearing potential (WOCBP) must agree to use at least one method of acceptable contraception (that is included in the table in this Appendix), from the time she signs consent, and until at

For purpose of this study, acceptable birth control methods that can achieve a failure rate of less than 1% per year when used consistently and correctly are considered as highly effective and include:

- Combined (estrogen and progestogen containing) hormonal contraception associated with inhibition of ovulation[1]:
  - oral
  - intravaginal
  - transdermal
- Progestogen-only hormonal contraception associated with inhibition of ovulation[1]:
  - oral
  - injectable
  - implantable[2]
- Intrauterine device (IUD)[2]
- Intrauterine hormone-releasing system (IUS)[2]
- Bilateral tubal occlusion[2]
- Vasectomised partner[2,3]
- Sexual abstinence[4]

For purpose of this study, acceptable birth control methods which may not be considered as highly effective that result in a failure rate of more than 1% per year include:

- Progestogen-only oral hormonal contraception, where inhibition of ovulation is not the primary mode of action
- Male or female condom with or without spermicide[5]
- Cap, diaphragm or sponge with spermicide[5]

---

[1] Hormonal contraception may be susceptible to interaction with the investigational medicinal product (IMP), which may reduce the efficacy of the contraception method.
[2] Contraception methods that in the context of this guidance are considered to have low user dependency.
[3] Vasectomised partner is a highly effective birth control method provided that partner is the sole sexual partner of the WOCBP trial participant and that the vasectomised partner has received medical assessment of the surgical success.
[4] In the context of this guidance sexual abstinence is considered a highly effective method only if defined as refraining from heterosexual intercourse during the entire period of risk associated with the study treatments. The reliability of sexual abstinence needs to be evaluated in relation to the duration of the clinical trial and the preferred and usual lifestyle of the subject.
[5] A combination of male condom with either cap, diaphragm or sponge with spermicide (double barrier methods) are also considered acceptable, but not highly effective, birth control methods.
Source: Clinical Trial Facilitation Group (CTFG), 2014.

least 30 days after her final dose of Pemigatinib.

A man able to father children who has a female partner of childbearing potential must agree to use at least one method of acceptable contraception (that is included in the table in this Appendix), from the time he signs consent, and until at least 90 days after his final dose of Pemigatinib .

A woman of childbearing potential participating in the study must have a negative serum pregnancy test during screening and a negative serum or urine pregnancy test on Cycle 1, Day 1 prior to her first dose of Pemigatinib.

- Women will be considered of childbearing potential (WOCBP), i.e. fertile, following menarche and until becoming post-menopausal unless permanently sterile. Permanent sterilization methods include hysterectomy, bilateral salpingectomy and bilateral oophorectomy.
- A post-menopausal state will be defined as no menses for 12 months without an alternative medical cause.

  A high follicle stimulating hormone (FSH) level in the post-menopausal range may be used to confirm a post-menopausal state in women not using hormonal contraception or hormonal replacement therapy. However in the absence of 12 months of amenorrhea, a single FSH measurement is insufficient and the medical judgment of the investigator will determine the number of FSH measurements greater than one (>1) which constitute a consistently elevated FSH level in the post-menopausal range.
- Male patients able to father children are defined as those who are not surgically sterile (i.e. patient has not had a vasectomy).

A trial physician or clinical designee shall counsel female patients of childbearing potential, and male patients able to father children who have a female partner of childbearing potential, regarding the importance of pregnancy prevention, the implications of an unexpected pregnancy, and the use of acceptable contraception. At a minimum, applicable patients must agree to the consistent and correct use of at least one method of acceptable contraception, as listed in the above table in this appendix.

Appendix II: Examples of CYP3A4 Inducers and Inhibitors

Please see http://medicine.iupui.edu/clinpharm/ddis/table.aspx for examples of CYP3A4 therapies. This list is not comprehensive. Contact your investigational/central pharmacy for any questions.

(Strong and moderate classifications may be referenced on Lexi-Comp Online, drug information reference http://online.lexi.com/crlsql/servlet/crlonline).

CYP3A4 INHIBITORS	
GENERIC NAME	TRADE NAME
Indinavir	Crixivan
Nelfinavir	Viracept
Ritonavir	Norvir
clarithromycin	Biaxin
itraconazole	Sporanox
ketoconazole	Nizoral
Nefazodone	Serzone
Saquinavir	Invirase
telithromycin	Ketek
Aprepitant	Emend
erythromycin	Ery-Tab
Fluconazole	Diflucan
grapefruit juice	
Verapamil	Calan
Diltiazem	Cardizem
Cimetidine	Tagamet
Amiodarone	Cordarone
chloramphenicol	Chloroptic
Delavirdine	Rescriptor
diethyldithiocarbamate	Imuthiol
fluvoxamine	Luvox
gestodene	Minesse
mibefradil*	Posicor
mifepristone	Mifeprex
norfloxacin	Noroxin
norfluoxetine	N/A
star fruit	
voriconazole	VFEND
amprenavir[1]	Agenerase
atazanavir[1]	Reyataz
fosamprenavir[1]	Lexiva
seville oranges	

CYP3A4 INDUCERS	
GENERIC NAME	TRADE NAME
efavirenz	Sustiva
nevirapine	Viramune
barbiturates	Luminal
carbamazepine	Tegretol
glucocorticoids	
modafinil	Provigil
nevirapine	Viramune
oxcarbazepine	Trileptal
phenobarbital	Luminal
phenytoin	Dilantin
pioglitazone	Actos
rifabutin	Mycobutin
rifampin	Rifadin
St. John's wort	
troglitazone*	Rezulin

Inducers	Therapeutic class
**Potent CYP3A Inducers**	
Rifampin	Antibiotics
Mitotane	Other Antineoplastics
Avasimibe	Other Antilipemics
Rifapentine	Antibiotics
Apalutamide	Antiandrogens
Phenytoin	Anticonvulsants
Carbamazepine	Anticonvulsants
Enzalutamide	Antiandrogens
St John’s Wort extract	Herbal medications
Lumacaftor	Cystic fibrosis treatments
Rifabutin	Antibiotics
Phenobarbital	Anticonvulsants
**Moderate CYP3A Inducers**	
Ritonavir and St. John’s wort	None
Semagacestat	Alzheimer’s treatments
Efavirenz	NNRTIs
Tipranavir and ritonavir	Protease inhibitors
Dabrafenib	Kinase inhibitors
Lesinurad	Antigout and uricosuric agents
Bosentan	Endothelin receptor antagonists
Genistein	Food products
Thioridazine	Antipsychotics
Nafcillin	Antibiotics
Talviraline	NNRTIs
Lopinavir	Protease inhibitors
Modafinil	Psychostimulants
Pf-06282999	Myeloperoxidase inactivators
Etravirine	NNRTIs
Lersivirine	NNRTIs
Telotristat ethyl	Antidiarrheals

CYP3A Inducers

Appendix III: ACCRU-GI-1701 SUBJECT MEDICATION DIARY

Name:	Subject ID Number:
Cycle:	

ORAL MEDICATION DIARY
Subject Instructions

- Please bring your Medication Diary and any empty or unused medication container(s) with you to every appointment
- Please use an ink pen when completing the Medication Diary as these will be retained in our research record.
- Please contact your physician and study coordinator any time you go into the hospital. Your physician can advise if you should stop taking your medication or continue it.
- To correct an error or mistake, please make a single line through that entry and write your initials and date next to the error or mistake.
- Please record each dose as soon as you take it and fill in the date as directed.
- Please indicate on the calendar below every day that you take your study medication by placing the dose taken on the line under the date.
- If you miss a dose, place a "0" under the date, but remember to take your prescribed dose at the next regularly scheduled time.
- If you accidentally take more than you are instructed to, contact your doctor or the emergency room immediately.
- If you miss a dose, do not make up the dose or double up the next dose.
- Your dose may be held due to side effects, be sure to record a 0 on those days your medication is held. Once you restart the medication, record the dose again.
- There will only be a maximum of 21 days of medication for each cycle recorded on this form

Example:

	**Day 1**	**Day 2**	**Day 3**	**Day 4**	**Day 5**	**Day 6**	**Day 7**
**Date**	1/1/2018	1/2/2018	1/3/2018	1/4/2018	1/5/2018	1/6/2018	1/7/2018
**Time**	7:00 AM						
Pemigatinib	Daily dose in mg						

ACCRU-GI-1701 SUBJECT MEDICATION DIARY – Pemigatinib

	**Day 1**	**Day 2**	**Day 3**	**Day 4**	**Day 5**	**Day 6**	**Day 7**
**Date**							
Time							
Pemigatinib Dose							

	**Day 8**	**Day 9**	**Day 10**	**Day 11**	**Day 12**	**Day 13**	**Day 14**
**Date**							
Time							
Pemigatinib Dose							

	**Day 15**	**Day 16**	**Day 17**	**Day 18**	**Day 19**	**Day 20**	**Day 21**
**Date**							
Time							
Pemigatinib Dose							

	**Day 22**	**Day 23**	**Day 24**	**Day 25**	**Day 26**	**Day 27**	**Day 28**
**Date**							
Time							
Pemigatinib Dose							

	**Day 29**	**Day 30**	**Day 31**	**Day 32**	**Day 33**	**Day 34**	**Day 35**
**Date**							
Time							
Pemigatinib Dose							

Date:__________________ Subject's Signature___________________________________

Area Below Only To Be Completed only by Coordinator

Week	1	2	3	4	5	Discrepancy (Yes or No)
Date of Pill Count						
Number of Pills Returned						
Study Coordinator Initials						

Appendix IV: ACCRU-GI-1701 SUBJECT INFORMATION SHEET
Subject Completed Quality of Life Booklet
(Baseline, Each Cycle, and end of treatment)

You will be given booklets to complete for this study. This booklet contains some questions about your 'quality-of-life' as a subject receiving treatment for cancer. Your answers will help us to better understand how the treatment you are receiving is affecting the way you feel.

1. You are being asked to complete a questionnaire booklet for this study. This booklet must be completed prior to dosing at each cycle and at the end of treatment (within 30 days after your last dose).

2. This booklet contains the following questionnaire:
   a. Linear Analogue Self Assessment (LASA)

3. Directions on how to complete each set of questions are written on the top of the page.

4. You will be given the nurse's or study coordinator's name and telephone number. You can call anytime with any concerns or questions.

5. Please complete the booklet and return it to your study staff. It is very important that you return the booklet to us.

Thank you for taking the time to help us.

Appendix V: ACCRU-GI-1701 LINEAR ANALOGUE SELF ASSESSMENT

Patient Name: ______________________________ Date: ____________________
Patient Number: ____________________________

Directions: Please circle the number (0-10) best reflecting your response to the following that describes your feelings **during the past week, including today**.

How would you describe:

1. your overall Quality of Life?

0	1	2	3	4	5	6	7	8	9	10
As bad as it can be										As good as it can be

2. your overall mental (intellectual) well being?

0	1	2	3	4	5	6	7	8	9	10
As bad as it can be										As good as it can be

3. your overall physical well being?

0	1	2	3	4	5	6	7	8	9	10
As bad as it can be										As good as it can be

4. your overall emotional well being?

0	1	2	3	4	5	6	7	8	9	10
As bad as it can be										As good as it can be

5. your level of social activity?

0	1	2	3	4	5	6	7	8	9	10
As bad as it can be										As good as it can be

6. your overall spiritual well being?

0	1	2	3	4	5	6	7	8	9	10
As bad as it can be										As good as it can be

7. the frequency of your pain?

0	1	2	3	4	5	6	7	8	9	10
No pain										Constant pain

8. the severity of your pain, on the average?

0	1	2	3	4	5	6	7	8	9	10
No pain										Pain as bad as you can imagine

9. your level of fatigue, on the average?

0	1	2	3	4	5	6	7	8	9	10
No fatigue										Constant Tiredness

10. your level of support from friends and family?

0	1	2	3	4	5	6	7	8	9	10
No support										Highest level of support

11. your financial concerns?

0	1	2	3	4	5	6	7	8	9	10
Constant concerns										No concerns

12. your legal concerns (will, advanced directives, etc.)?

0	1	2	3	4	5	6	7	8	9	10
Constant concerns										No concerns

© 1996 Mayo Foundation for Medical Research
